# The genome and transcriptome of the enteric parasite *Entamoeba invadens*, a model for encystation

**DOI:** 10.1186/gb-2013-14-7-r77

**Published:** 2013-07-26

**Authors:** Gretchen M Ehrenkaufer, Gareth D Weedall, Daryl Williams, Hernan A Lorenzi, Elisabet Caler, Neil Hall, Upinder Singh

**Affiliations:** 1 Division of Infectious Diseases, Department of Internal Medicine, Stanford University, Stanford, California, 94305, USA; 2 Institute of Integrative Biology, University of Liverpool, Crown Street, Liverpool, UK; 3 J Craig Venter Institute, Rockville, Maryland, L697ZB USA; 4 Faculty of Science, King Abdulaziz University, Jeddah, 21589, SA; 5 Department of Microbiology and Immunology, Stanford University, Stanford, California, 94305, USA

## Abstract

**Background:**

Several eukaryotic parasites form cysts that transmit infection. The process is found in diverse organisms such as *Toxoplasma*, *Giardia*, and nematodes. In *Entamoeba histolytica *this process cannot be induced *in vitro*, making it difficult to study. In *Entamoeba invadens*, stage conversion can be induced, but its utility as a model system to study developmental biology has been limited by a lack of genomic resources. We carried out genome and transcriptome sequencing of *E. invadens *to identify molecular processes involved in stage conversion.

**Results:**

We report the sequencing and assembly of the *E. invadens *genome and use whole transcriptome sequencing to characterize changes in gene expression during encystation and excystation. The *E. invadens *genome is larger than that of *E. histolytica*, apparently largely due to expansion of intergenic regions; overall gene number and the machinery for gene regulation are conserved between the species. Over half the genes are regulated during the switch between morphological forms and a key signaling molecule, phospholipase D, appears to regulate encystation. We provide evidence for the occurrence of meiosis during encystation, suggesting that stage conversion may play a key role in recombination between strains.

**Conclusions:**

Our analysis demonstrates that a number of core processes are common to encystation between distantly related parasites, including meiosis, lipid signaling and RNA modification. These data provide a foundation for understanding the developmental cascade in the important human pathogen *E. histolytica *and highlight conserved processes more widely relevant in enteric pathogens.

## Background

Conversion between distinct developmental stages is an essential part of the life cycle of many pathogens and is necessary for transmission. For enteric protozoa, the transmissible stage is the cyst, which allows survival outside of the host [1]. Understanding the molecular processes controlling stage conversion is central to the development of transmission-blocking therapies as well as novel diagnostics [2,3]. *Entamoeba histolytica *causes colitis and dysentery and infects 500 million people per year worldwide [4]. The related *Entamoeba invadens *causes a similar invasive disease in reptiles [5]. The *Entamoeba *life cycle has two stages: trophozoites, which proliferate in the colon and cause disease, and non-dividing, multinucleate cysts that are transmitted to new hosts [6].

Research into the molecular basis of conversion between these two forms has been hampered by the absence of tools to induce encystation and excystation in *in vitro *axenic cultures of *E. histolytica *[7,8]. Clinical *E. histolytica *isolates maintained in xenic culture are capable of stage interconversion and have been used to examine the transcriptome of *E. histolytica *cysts [9]. However, the percentage of cells forming cysts is low and stage conversion is asynchronous [10]. While interesting developmentally regulated genes were identified, the inability to isolate cysts at different developmental stages likely prevented the discovery of many important regulators of encystation.

Due to the lack of *in vitro *methods for studying encystation in *E. histolytica*, the reptile parasite *E. invadens *has been utilized as a model system to study development. The IP-1 strain was originally isolated from a natural infection of a painted turtle, *Chrysemys picta*, and is pathogenic in snakes [5]. *E. invadens *IP-1 can form cysts in axenic culture and methods have been developed to induce high efficiency encystation and excystation *in vitro *[6,11]. Using this system, many features of cyst wall biosynthesis have been elucidated [12,13] and several compounds that enhance or inhibit encystation have been identified, including protein kinase C inhibitors and cytochalasins [14-16], suggesting that these pathways may be involved in regulating development. Recently, genetic tools have been developed to allow stable protein expression in *E. invadens *[17,18], further enhancing its usefulness as a model system.

Genome-wide transcriptional profiling using microarrays has been an important tool for increasing our understanding of parasite stage conversion [19-21]. Recent advances in high-throughput sequencing have allowed development of RNA-Sequencing (RNA-Seq), in which an entire transcriptome (reviewed in [22]) is sequenced and relative expression of each transcript deduced from read frequencies. In this paper we present the genome assembly and annotation of *E. invadens *IP-1, RNA-Seq analysis of transcriptional changes during the complete developmental cycle (encystation and excystation), and the functional demonstration that perturbation of the phospholipase D (PLD) pathway inhibits stage conversion in *Entamoeba*. Our findings demonstrate major changes in gene expression during encystation and excystation in *Entamoeba*, and provide insight into the pathways regulating these processes. A better understanding of processes regulating stage conversion may guide targeted interventions to disrupt transmission.

## Results and discussion

### The *E. invadens *genome assembly and predicted gene models

**Figure 1:**
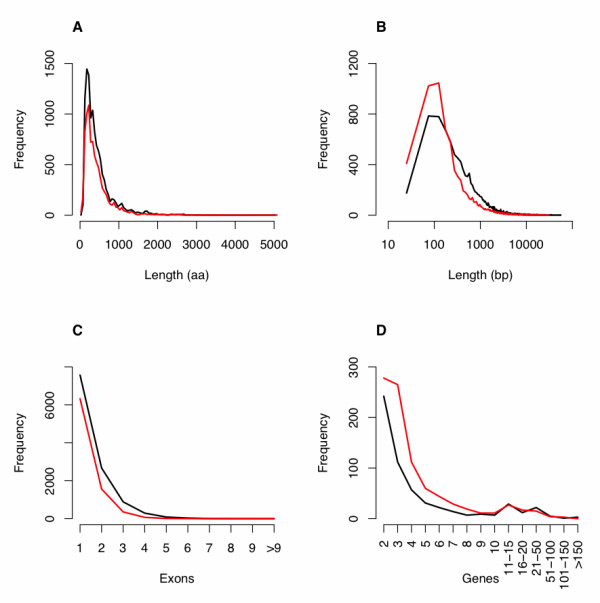
**Comparisons of protein length, intergenic region length, exon number and gene family size in the *E*. *invadens *and *E. histolytica *genomes**. In all plots, the black line represents *E. invadens *and the red line represents *E. histolytica*. **(a) **Distribution of protein lengths for the translated coding sequences. **(b) **Distribution of intergenic sequence lengths. For display purposes, the x-axis is plotted in a log scale. **(c) **Distribution of the number of exons per gene. **(d) **Distribution of the number of putative paralogous genes belonging to multi-gene families. Gene families were defined based on shared functional domains (see Materials and methods for detailed description).

In order to determine the genome sequence of *E. invadens*, 160,419 paired-end Sanger sequenced reads derived from *E. invadens *genomic DNA were assembled [23]. A small number of contigs were removed due to small size and possible contamination, and a total of 4,967 contigs in 1,144 scaffolds were submitted to GenBank under the accession number [AANW00000000] (Bioproject PID PRJNA12926). The total scaffold span (including estimated gap sizes) was 40,878,307 bp (40,496,007 bp excluding gaps). The average intra-scaffold gap size was estimated to be 660 bases (however, all gaps were represented by 100 'N's in the final assembly). Over 50% of the assembly is represented in scaffolds larger than 231,671 bases and contigs larger than 17,796 bases. The total assembly size was nearly twice that of *E. histolytica *(approximately 22 MB). The nucleotide composition (35% A, 35% T, 15% G, 15% C) was slightly less A+T-rich than *E. histolytica *(70% versus 75% A+T). Automated gene prediction and manual curation defined 11,549 putative protein coding genes analyzed in this study (the number of genes in the GenBank genome was higher at 11,997, due to genes containing gaps being split into two or more genes). The predicted protein length distribution is shown in Figure 1a. Of these gene models, 35% were predicted to contain one or more intron (Table 1).

**Table 1:** Introns detected by whole transcriptome mapping

Sample	Introns	Confirm annotation (%)^a^	Match 5'	Match 3'	Novel
Trophozoite	1,628	1,345 (23%)	19	11	253
Cyst_8h	2,163	1,771 (30%)	23	10	359
Cyst_24h	1,955	1,536 (26%)	21	12	386
Cyst_48h	1,047	811 (14%)	15	5	216
Cyst_72h	1,272	1,027 (17%)	9	6	230
Excyst_2h	1,438	1,229 (21%)	12	6	191
Excyst_8h	764	666 (11%)	5	2	91
All	3,239	2,470 (42%)	35	17	717

**^a^**Percentage of 5,894 introns predicted from genome annotation.

Of the 11,549 predicted *E. invadens *genes, 9,865 have a BLASTP (E-value <10^-5^) hit to an *E. histolytica *gene (7,216 of the 8,306 predicted genes in *E. histolytica *had a BLASTP hit to an *E. invadens *gene) and 5,227 genes were putative orthologs (reciprocal best BLAST hits). Average amino acid identity between aligned regions of orthologs is 69%, suggesting that the species are distantly related (similar in distance to *Plasmodium falciparum *and *Plasmodium vivax*, for example). Of the *E. invadens *genes without orthologs in *E. histolytica*, 77% (4,815/6,218) have at least some RNA-Seq support, compared to 98% (5,206/5,331) of genes shared with *E. histolytica*. This result could suggest that a proportion of these genes are false positive predictions; however, it is also consistent with these being contingency genes that are not constitutively expressed so transcripts are less likely to be detected.

To identify the level of conserved synteny between the two species, we identified all collinear gene pairs that were adjacent in both *E. histolytica *and *E. invadens*. Only 561 genes maintained their neighboring gene in both species (out of a total of 5,227). Hence, it appears that there has been extensive genomic rearrangement between these species.

Both *E. histolytica *and *E. invadens *genomes are highly repetitive and only around 50% of the genome size, in both species, is accounted for by genic and intergenic sequence due to the large number of contigs that are unscaffolded and do not contain annotation. The larger genome size of *E. invadens *cannot be accounted for simply by the greater number of predicted genes: 11,549 in *E. invadens *compared to 8,306 in *E. histolytica*. We compared the length distributions of genes and intergenic sequence in the two genomes; Figure 1 shows the distribution of gene and intergenic sizes in the two species. It is clear from these analyses that the gene lengths of *E. histolytica *and *E. invadens *are very similar whereas the intergenic regions in *E. invadens *tend to be longer than those in *E. histolytica*. A previous analysis of transposons and retrotransposons in *E. invadens *[24] suggests that repetitive elements are not more common in *E. invadens*. Thus, the longer intergenic regions are unlikely to have increased in size due to transposon/retrotransposon activity, as the previous analysis failed to identify many *E. invadens*-specific repeat elements. However, one possibility is that differences in annotation and the lower depth of coverage in *E. invadens *resulted in an under-calling of genes, thus making intergenic regions appear larger in *E. invadens*. To check this, we compared the sizes of the intergenic spaces between the 561 pairs of colinear orthologous genes identified in the syntenic analysis. This revealed that the mean intergenic distance between gene pairs in *E. invadens *is 408 bp while it is only 282 bp in *E. histolytica*. In both *E. histolytica *and *E. invadens *the mean distance between genes where they were divergently transcribed (550 bp and 341 bp) was on average, considerably larger than the distance between genes that were transcribed toward each other (284 bp and188 bp), presumably because in both species the 5' regions were required for transcription factor binding. Considered together, these observations suggest an expansion of the intergenic regions in *E. invadens *relative to *E. histolytica*, possibly as a result of differential strengths of selection on intergenic sequence size - for example, weaker selection against expansion in *E. invadens *may allow intergenic regions to expand through genetic drift. However, in some fungal plant pathogens genome expansion has been associated with adaptation to different hosts [25], as gene family expansion and repeat driven chromosomal rearrangement can accelerate genomic diversity. As *E. invadens *infects a broad range of hosts, including lizards, snakes and turtles, while *E. histolytica *is primarily associated with humans and primates, it is possible that the observed difference in genome size reflects this discrepancy of host range restriction.

The genome of *E. histolytica *is highly repetitive, with many genes occurring in large multi-gene families [24]. This is also the case in *E. invadens*. Predicted proteins were clustered into putative gene families based on possession of shared domains (see Materials and methods section and Additional file 1 for description). There were 572 families of 2 or more genes and 78 families of 10 or more genes. The distribution of gene family sizes is shown in Figure 1d and 1all genes assigned to multigene families are shown in Additional file 2. The predicted functions of the largest gene families highlight the importance of motility and signaling in the organism's survival. The largest gene families include two families of protein kinases (*n *= 410 and *n *= 135), phosphatases (*n *= 74), small GTP-binding proteins (*n *= 225), Rho-GTPases (*n *= 84), Rho/Rac guanine nucleotide exchange factors (*n *= 41), calcium-binding proteins (*n *= 70), WD-repeat-containing proteins (*n *= 61), actins (*n *= 53) and RNA-binding proteins (*n *= 48).

In addition to these well characterized gene families, the *E. invadens *genome contains representatives of gene families recently identified as having important biological roles in *E. histolytica*, including RNA interference pathway genes and Myb domain-containing transcription factors [26-29]. RNA interference (RNAi) is an important mechanism for gene regulation that has been found in the majority of eukaryotes studied [30]. Recently, the existence of an active RNAi pathway has been demonstrated in *E. histolytica *and found to be involved in gene silencing and strain-specific gene expression patterns [26,31,32]. In *E. histolytica*, small RNAs map to a subset of genes that are not expressed in trophozoites but are expressed in cysts [31], suggesting that RNAi could help regulate development in *Entamoeba*. Argonaute, a vital member of the RNA-induced silencing complex [33], is characterized by two conserved domains: the PAZ domain, which enables binding of small RNAs, and the PIWI domain, which is thought to be important for RNA cleavage. Examination of the *E. invadens *genome indicated the presence of two full-length Argonaute proteins (EIN_033570 and EIN_035430), a single PAZ domain protein (EIN_182430) and a PIWI domain protein (EIN_182370). Additionally, the *E. invadens *genome contains genes encoding RNA-dependent RNA polymerase (EIN_181590 and EIN_092660), thought to be required for the formation of small RNAs, and a single RNAseIII domain-containing gene (EIN_108010). The presence of these RNAi pathway genes in *E. invadens *suggests that an endogenous small RNA pathway may also regulate gene expression in *E. invadens*.

Myb family transcription factors are important regulators of gene expression. Although originally identified in mammals, where they play important roles in cell proliferation and differentiation [34], Myb domain-containing proteins have subsequently been identified in diverse species [35,36], and they are the largest family of transcription factors in *E. histolytica *[37]. *E. histolytica *Myb domain-containing proteins have been implicated in development [29] and in the response to oxidative stress [27]. Myb proteins have also been shown to be regulated during colonic invasion [38] and liver abscess formation [39], indicating that these proteins are important in multiple aspects of amebic biology, and suggesting that this genus-specific expansion is required for *Entamoeba*-specific functions. We identified 44 Myb domain-containing proteins in the *E. invadens *genome, including 9 that contain a conserved SHAQKY motif (EIN_053790, EIN_054250, EIN_059020, EIN_134750, EIN_241140, EIN_020200, EIN_277760, EIN_015270, EIN_051670), indicating they are members of a sub-family of Myb proteins. This family is common in plants [40] and is found in *Dictyostelium*, where a SHAQKY domain protein was shown to regulate pre-stalk cell genes [41]. Further investigation will be required to elucidate potential roles for these proteins in biological processes of *Entamoeba *such as stage conversion.

Despite the different size of the *E. invadens *genome, our analysis suggests that it is very similar to *E. histolytica *in its core gene content. Although there has been lineage-specific expansion of intergenic regions and some gene families, the large family of Myb transcription factors and the machinery for RNAi has been conserved, suggesting that *E*. *invadens *is a good model for expression analysis.

### Whole transcriptome mapping to the *E. invadens *genome assembly

**Figure 2:**
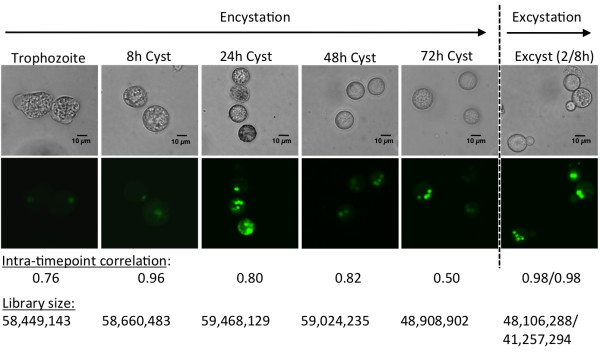
**Changes in cell morphology during encystation and excystation**. Cell morphology at different time points during encystation and excystation. The top row shows representative phase contrast images; the bottom row shows cells stained with Syto-11 to stain the nuclei. Note that the numbers of nuclei vary between one and four per cell, indicating some heterogeneity among cells in the timing of events during encystation, with the majority of mature cysts with four nuclei. Scale bar indicates 10 µm. Total library size and Pearson correlation between biological replicates is shown for each time point.

In order to understand changes in gene regulation during *E. invadens *stage conversion and to assess the genome annotation, the transcriptomes of encysting and excysting parasites were sequenced. *E. invadens *trophozoites were induced to encyst by incubation in 47% low glucose media [42], and RNA was generated from 0 h, 8 h, 24 h, 48 h, and 72h time points (with the 72 h time point representing mature cysts). The experimental design is outlined in Figure 2. Samples from excysting parasites were generated by harvesting mature cysts (72 h after induction of encystation), incubating overnight in distilled water to eliminate any remaining trophozoites, and transferring to excystation medium [11] for 2 h or 8 h. Only samples with high encystation or excystation efficiencies (>80% encystation assayed at 72 h or >60% excystation assayed at 24 h) were used for RNA analysis.

For each time point during encystation and excystation, short read sequencing libraries were generated from cDNA from two independent biological replicates. Libraries were sequenced on a SOLiD™ 4 sequencer, and aligned to the *E. invadens *genome assembly [43]. Mapping statistics (Additional file 3) revealed that the proportion of sequences that aligned to the reference genome was comparable to published data [44]. The unmapped proportion of each library was only partially (<5%) accounted for by tRNA gene arrays or rDNA genes, which are not represented in the genome assembly. Overall, reads that mapped to the genome were of high quality (>30 phred score for all samples tested), giving further confidence that the mappings are valid. The correlation between biological replicates at each encystation and excystation time point (read counts per gene are shown in Additional file 4, read count correlations are shown in Additional file 5) revealed that replicates correlated to a reasonable degree, although some disparities were identified. Given that the encystation process is asynchronous, stochastic biological variation likely accounts for the differences. This variation among samples will make it difficult to identify subtle changes in gene expression but differential expression of more highly regulated genes can still be identified, given statistical significance [45], and provide important biological insights.

### Assessment of the accuracy of predicted *E. invadens *gene models using transcriptome data

Mapping of RNA-Seq reads identified many unannotated transcribed regions of the genome. Many of these may be transcribed transposable elements but some may represent unannotated protein coding genes. In order to detect these, we mapped the transcriptome data to the genome using Tophat v1.3.2 [46], determined putative transcripts using Cufflinks and selected those that did not overlap an annotated gene. We then translated their sequences and used these to search for functional protein domains in the Pfam database [47]. The results are shown in Additional file 6. Common domains included DDE_1 transposases that are associated with DNA transposons, and hsp70 domains. In general, unannotated transcripts did not contain a single long open reading frame, indicating that genes were not predicted due to being pseudogenes or artifacts of low sequence coverage of the genome assembly. Overall, we did not find evidence of numerous long un-annotated open reading frames that had been missed by automated gene prediction.

**Figure 3:**
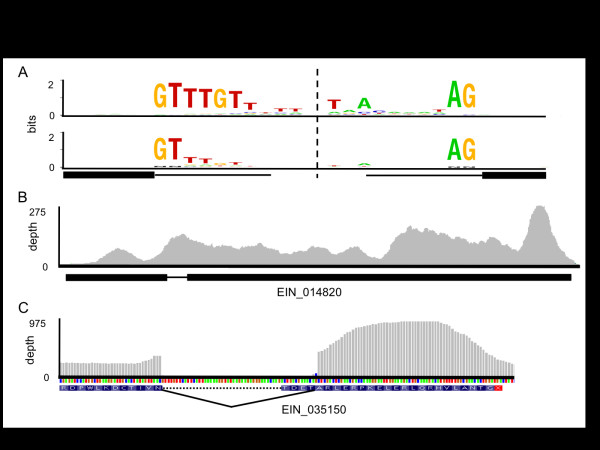
**Analysis of *E*. *invadens *introns**. **(a) **Sequence logos representing consensus sequences at 5' and 3' intron-exon junctions for introns detected by whole transcriptome mapping (upper) and for introns predicted by computational gene prediction but not confirmed by transcriptome mapping (lower). The start and end of the intron are indicated by the thick black line and intronic sequence by the thin line. The dashed line indicates that the central portions of the introns were not analyzed. **(b) **Example of a probably incorrect gene model (EIN_014820, thick black line) that contains an intron (thin black line) that is contradicted by transcriptome mapping (depth of coverage shown in grey plot). **(c) **Example of probably incorrect gene model (EIN_035150, part shown with predicted translation) in which the predicted intron (dashed line) is supported by whole transcriptome mapping at the 5' junction only. The correct intron is indicated by the solid black line. The corrected protein is shorter by four amino acids (TDET).

To assess the accuracy of the genome annotation, we used the transcriptome data (mapped with Tophat v1.3.2 [46]) to identify introns. Overall, the alignment identified 3,239 putative introns; 2,470 of these were among the 5,894 predicted by computational gene calling (Additional file 7). A further 52 matched a predicted intron at only the 5' (35) or 3' (17) end, indicating a small number of mis-annotated introns (example in Figure 3c). A proportion of the 3,424 non-confirmed introns may be annotation errors, as suggested by a difference between the 5' consensus sequence of confirmed and non-confirmed introns (Figure 3a). Confirmed introns show an extended 5' consensus sequence (GTTTGT) compared to only the GT in unconfirmed introns, a pattern also seen in *E. histolytica *introns [48,49]. Other non-confirmed introns contained sequencing gaps ('N' bases in the scaffold sequence), which might cause artifacts (introns that span a gap to make an intact gene model) in computational gene calling. Although these only accounted for 13.6% (464/3,424) of the non-confirmed introns, this proportion was much higher than the 0.1% (2/2,470) of confirmed introns that had sequencing gaps. To determine where the transcriptome data contradicted a predicted intron, we counted the number of 35 bp reads that mapped entirely within each predicted intron. Overall, 308 predicted but non-confirmed introns had more than five reads (from all libraries combined) aligned in the predicted intron (example shown in Figure 3b). However, we also identified 276 cases in which an intron was both confirmed and had >5 reads mapped within it. Whether this indicates intron retention in the transcripts, antisense transcripts, or low-level genomic DNA contamination is uncertain. Therefore, we could not use this to reject a predicted intron. In a small number of cases, the intron changed the reading frame of the gene model, or appeared to differ among libraries. This could be due to alternative splicing, or could be a reflection of stochastic noise, as recently observed in *E. histolytica *[49]. Overall, the transcriptome data provide empirical evidence confirming approximately 42% of the predicted introns in the genome.

### Changes in gene expression during encystation and excystation

To explore transcriptional changes during encystation and excystation we estimated gene expression levels of the 11,549 putative protein coding genes at time points during encystation (0 h, 8 h, 24 h, 48 h and 72 hours after induction of encystation) and excystation (2h and 8 h after induction of excystation). Normalized expression values for all genes were calculated (as fragments per kilobase of transcript per million mapped fragments (FPKM)) using Cufflinks v1.3.2 [46] (all shown in Additional file 4). The majority of genes (87%; 9,992/11,549) were expressed at at least one time point, with between 55% (at 48 h encystation) and 78% (at 2h excystation) expressed at any one time point. Expression levels were compared using two methods: (1) clustering genes by their temporal expression profile during encystation and excystation to gain a broad overview of transcriptional changes; (2) statistical pairwise comparisons of all time points to identify significantly up- and down-regulated genes.

**Figure 4:**
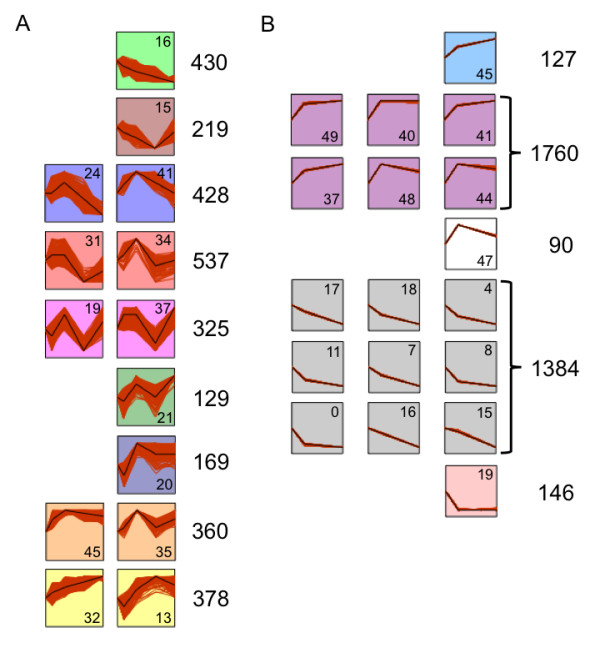
**Temporal gene expression profiles**. Temporal gene expression profiles containing significantly more genes than expected by chance. **(a) **Expression profiles during encystation. **(b) **Expression profiles during excystation. Each box shows a representative expression profile (black line) and profiles of genes assigned to the profile (red lines). The profile ID number is shown in each box. Clusters of related profiles have the same color. The numbers of genes assigned to a profile/cluster are shown to the right. All profiles are shown in Additional file 8 and data for all genes are tabulated in Additional file 4.

We defined temporal profiles of gene expression during encystation (0 h, 8 h 24 h, 48 h and 72 hours post-encystation) and excystation (72 hour cysts, 2 h and 8 h post-excystation), for 4,577 and 5,375 genes expressed at all time points in each series, using the short time-series expression miner (STEM) program [50]. All temporal expression profiles are shown in Additional file 8, and genes belonging to each profile are tabulated in Additional file 4. Nine clusters of related profiles contained significantly more genes than expected by chance during encystation and five similarly enriched clusters during excystation (Figure 4). During encystation, profiles showing general down-regulation over time were significantly enriched for proteins associated with translation and ribosome assembly Gene Ontology (GO) terms, while profiles showing up-regulation were significantly enriched for nuclear proteins associated with nucleosome assembly (Table 2). In general, the reverse trend was seen during excystation (Table 3). The results indicate a broad shift from active vegetative growth and protein production to a quiescent form with 'packaged' DNA in cysts. No consistent enrichment for GO terms was seen for encystation profiles peaking at 8 h or 24 h.

**Table 2:** Gene Ontology terms associated with temporal gene expression profiles during encystation

Profile/cluster^a^	GO category name	Fold enrichment^b^	Corrected *P*-value^c^
Profile 16	Translation	7.8	<0.001
	Structural constituent of ribosome	7.9	<0.001
	Ribosome	8.1	<0.001
	Protein binding	2.9	<0.001
	Actin binding	4.9	<0.001
	Glycolysis	12.2	<0.001
	Intracellular	1.7	<0.001
	Cytoplasm	4	0.002
	Catalytic activity	2.3	0.002
	Oxidoreductase activity	3.4	0.002
	Oxidation-reduction process	4	0.038
Profile 15	Translation	12.1	<0.001
	Structural constituent of ribosome	11.8	<0.001
	Ribosome	11	<0.001
	Small ribosomal subunit	24.3	<0.001
	Intracellular	2.2	<0.001
	Aminoacyl-tRNA ligase activity	16.6	<0.001
	tRNA aminoacylation for protein translation	16.6	<0.001
	Cytoplasm	6.5	<0.001
	NAD binding	16.5	<0.001
	Metabolic process	3	0.018
Cluster (profiles 24, 41)	Heat shock protein binding	6.7	0.004
Profile 24	Heat shock protein binding	8.5	0.006
	Unfolded protein binding	6.3	0.01
	Protein folding	5.7	0.014
Cluster (profiles 31, 34)	Metabolic process	3.4	<0.001
	Catalytic activity	2.5	<0.001
	Binding	2.3	<0.001
	Phosphatidylcholine-sterol O-acyltransferase activity	9	0.016
	Membrane coat	5.8	0.016
	Structural molecule activity	6.8	0.018
	Vesicle-mediated transport	3.3	0.04
Profile 31	Metabolic process	3.5	<0.001
	Catalytic activity	2.6	<0.001
	Binding	2.4	<0.001
	Membrane coat	6.8	0.002
	Phosphatidylcholine-sterol O-acyltransferase activity	10.5	0.002
	Structural molecule activity	7.9	0.002
	Vesicle-mediated transport	3.9	0.004
	Lipid metabolic process	4.9	0.034
Profile 19	Vesicle-mediated transport	5.1	0.062
	Nucleic acid binding	2.2	0.464
Profile 21	Integral to membrane	4.7	0.014
	Membrane	4.1	0.038
Profile 20	Hydrolase activity	4	0.014
Cluster (profiles 32 13)	Methionine adenosyltransferase activity	30.5	<0.001
	One carbon metabolic process	30.5	<0.001
	DNA binding	3.6	<0.001
	Nucleosome	12.2	0.002
	Nucleus	3	0.002
	Nucleosome assembly	8	0.002
	Sequence-specific DNA binding	6.9	0.034
Profile 32	Methionine adenosyltransferase activity	48.7	<0.001
	One carbon metabolic process	48.7	<0.001
	DNA binding	3.8	<0.001
	Nucleus	3.3	0.01
Profile 13	Intracellular protein transport	5.3	0.002
	Intracellular	2.3	0.002
	GTP binding	3.3	0.002
	Small GTPase mediated signal transduction	3.1	0.002
	Protein transport	3.4	0.004
	Nucleocytoplasmic transport	5.6	0.016
	GTPase activity	4.3	0.036

^a^See Figure 4 and Additional file 8 for profiles/clusters. ^b^Ratio of the observed number of genes with associated GO term to the number expected for a profile of the same size. ^c^*P*-value Bonferroni-corrected for multiple tests.

**Table 3:** Gene Ontology terms associated with temporal gene expression profiles during excystation

Profile/cluster^a^	GO category name	Fold enrichment^b^	Corrected *P*-value^c^
Profile 45	Protein binding	3.1	0.002
	Catalytic activity	3.4	0.002
	Metabolic process	3.4	0.05
Cluster (profiles 49, 40, 41, 37, 48, 44)	Zinc ion binding	1.7	<0.001
	Catalytic activity	1.7	<0.001
	Pyridoxal phosphate binding	3.7	0.024
	Nucleic acid binding	1.6	0.028
	Heat shock protein binding	3	0.032
Profile 49	Helicase activity	4.3	0.042
	Ubiquitin-dependent protein catabolic process	4.1	0.05
Profile 40	Binding	2.1	0.01
	Zinc ion binding	2	0.038
Profile 41	Membrane	3.7	0.006
Profile 37	Protein binding	2.4	0.022
	Catalytic activity	2.5	0.048
Profile 47	Oxidoreductase activity	6.3	0.018
Cluster (profiles 0, 4, 7, 8, 11, 15, 16, 17, 18)	Ribosome	3.7	<0.001
	Structural constituent of ribosome	3.6	<0.001
	Translation	3.1	<0.001
	Intracellular	1.5	<0.001
	DNA binding	2.1	<0.001
	Nucleosome	5.6	<0.001
	Nucleus	2.1	<0.001
	Nucleosome assembly	4	0.002
	Signal transduction	1.6	0.024
	Threonine-type endopeptidase activity	4.2	0.044
	Proteasome core complex	4.2	0.044
	Proteolysis involved in cellular protein catabolic process	4.2	0.044
Profile 17	Ribosome	7.1	<0.001
	Structural constituent of ribosome	6.8	<0.001
	Translation	6.3	<0.001
	Intracellular	1.9	0.002
Profile 18	Intracellular	2.1	0.006
	Ribosome	5	0.006
	Structural constituent of ribosome	4.8	0.008
	Translation	4.2	0.02
	Signal transduction	3	0.024
Profile 7	Ribosome	5.3	0.002
	Structural constituent of ribosome	5.1	0.002
	Translation	4	0.012
	DNA binding	3.1	0.04
Profile 8	Intracellular	2.3	0.01
	Protein transport	3.6	0.012
	GTP binding	3	0.024
	Catalytic activity	3.2	0.024
	Small GTPase mediated signal transduction	2.9	0.032
Profile 16	DNA binding	4.4	<0.001

^a^See Figure 4 and Additional file 8 for profiles/clusters. ^b^Ratio of the observed number of genes with associated GO term to the number expected for a profile of the same size. ^c^*P*-value Bonferroni-corrected for multiple tests.

**Figure 5:**
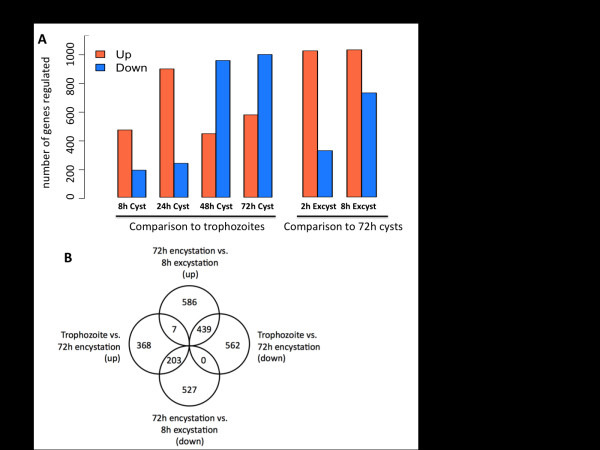
**Genes up-regulated and down-regulated in pairwise comparisons**. **(a) **Total number of genes significantly up- or down-regulated (as determined by Cuffdiff) in 8 h, 24 h, 48 h and 72 h encystation compared to trophozoites, and 2 h and 8 h excystation, compared to 72 h cysts, is shown. **(b) **Venn diagram showing overlap between the sets of genes up- or down-regulated in 72 h cysts compared to trophozoites, and genes up- or down-regulated in 8 h excysting parasites compared to 72 h cysts.

In addition to the temporal expression profiles, significantly differentially expressed genes (false discovery rate (FDR) <0.01) were identified from each pairwise comparison, using Cuffdiff [51] (all FPKM values and significantly differentially expressed genes are shown in Additional file 9). Strikingly, the numbers of genes up- and down-regulated at different time points varied greatly (Figure 5a). In early encystation (8 to 24 h) many genes were up-regulated when compared to trophozoites (472 and 900 genes, respectively), but fewer genes were down-regulated (190 and 238 genes, respectively). Later in encystation, this pattern reversed, with more genes down-regulated in 48 and 72 h cysts (959 and 1,001 genes, respectively) than up-regulated (446 and 578 genes, respectively), relative to trophozoites. During excystation, transcription of many genes is reactivated, with 1,025 genes being up-regulated at 2 h and 1,032 genes up-regulated at 8 h (compared to 72 h cysts) and comparatively fewer genes down-regulated (325 genes and 730 genes, at 2 h and 8 h, respectively). In general, trends in transcription during encystation are reversed during excystation (Figure 5b). The transcriptional changes during encystation suggest a developmental program activated in early cysts that is later turned off, and down-regulation of genes involved in general metabolic processes as cysts mature; transcription of these genes then resumes during excystation. Overall, approximately half of all *E. invadens *genes (6,168/11,549 total genes) were significantly differentially expressed at at least one time point. This scale of change in the transcriptome has been reported in *Plasmodium *[19] and *Leishmania *[52] development, though it sharply contrasts with findings in *Giardia lamblia *[53], where an extremely limited set of genes showed altered expression during encystation. These differences may indicate variances in the degree to which gene expression at the level of transcription or RNA stability regulates biological processes in these organisms.

**Figure 6:**
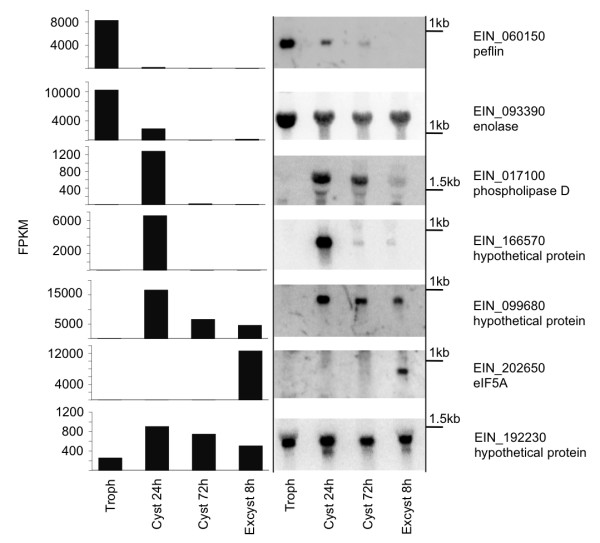
**Confirmation of transcript expression levels by Northern blot analysis**. Northern blot analysis of samples from trophozoites, at 24 and 72 h post-encystation, and at 8 h post-excystation. Total RNA (20 µg) from each time point was used and expression levels of genes with regulated expression determined. For each probe, the gene being probed and its annotation are listed. A gene with no significant changes in gene expression (EIN_192230) was used as a loading control. The expression level (FPKM) at each time point included in the blot is shown for each gene.

RNA-Seq results were confirmed for selected genes by Northern blot analysis of RNA isolated from trophozoites, 24 h encysting parasites, 72 h cysts and 8 h excysting parasites (Figure 6). Two genes with higher expression in trophozoites (EIN_060150, EIN_093390), three genes with higher expression during encystation (EIN_017100, EIN_166570 and EIN_099680), and one gene with increased expression in excystation (EIN_202650) were tested, confirming the patterns of expression identified by RNA-Seq. A gene with stable expression at all time points (EIN_192230) was used as a control. That RNA was derived from different biological samples from those used for RNA-Seq indicates the robustness of the regulation and the reliability of the RNA-Seq results.

### Comparison to previous *Entamoeba *development datasets

**Figure 7:**
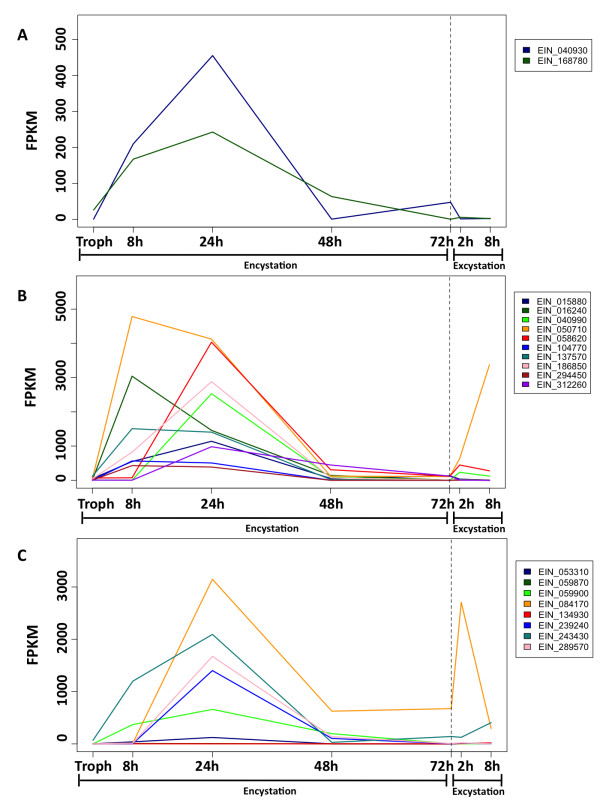
**Changes in the estimated expression levels of known encystation associated genes**. Expression levels (FPKM) are plotted for each time point during encystation and excystation. Data for all time points used in this study (trophozoite, 8 h, 24 h, 48 h, and 72 h encystation and 2 h and 8 h excystation) are shown. Dotted line indicates transition between encystation and excystation. **(a) **Expression of genes encoding chitin synthase domain-containing proteins. **(b) **Expression of genes encoding chitin binding lectins. **(c) **Expression of genes encoding chitinase domain-containing proteins

We analyzed the expression of genes previously identified as developmentally regulated in *Entamoeba*. As expected, genes encoding proteins involved in cyst wall synthesis are highly regulated during development, although interestingly different gene families within this category show distinctive patterns of expression. While the two identified chitin synthase family genes (EIN_040930 and EIN_168780) have increased expression by 8 h in encystation media (Figure 7a), the chitin binding lectins that form the protein component of the cyst wall [54] show varying patterns of expression, with many genes not induced until 24 h (Figure 7b). Interestingly, a chitinase domain containing protein, EIN_084170, was strongly up-regulated during excystation, suggesting it could be involved in parasite exit from the cyst (Figure 7c). In addition to these cyst-specific genes, EHI_148790, a member of the gene family of light chain subunits of the amoebic Gal/GalNac lectin, an important virulence factor in *E. histolytica *[55], was previously identified as being trophozoite specific in *E. histolytica *[9]; the putative *E. invadens *ortholog, EIN_281690 (60% amino acid identity), was significantly down-regulated in mature (48 to 72 h) cysts compared to trophozoites.

Overall, there was significant overlap between genes identified as developmentally regulated in the current RNA-Seq analysis and our previous study of the *E. histolytica *cyst transcriptome [9]. For the 393 genes up-regulated in *E. histolytica *cysts that had identifiable *E. invadens *homologs, 90 of the *E. invadens *genes were found to be up-regulated in at least one encystation time point (*P *≈ 1.4 × 10^-7^). Additionally, 93 genes were up-regulated at 2 or 8 h post-excystation (*P *≈ 2.5 × 10^-10^), likely due to the fact that the *E. histolytica *cyst transcriptome analysis was performed using an asynchronous population, including both encysting and excysting cells.

Recently, two papers comparing *Entamoeba *cysts and trophozoites have been published: a proteome of *E. histolytica *cysts isolated from patient samples [3] and a metabolomic study of encysting *E. invadens *[56], which reported expression data for a limited number of genes involved in metabolism. Although both studies were limited in scope (417 cyst proteins identified in *E. histolytica*, and 127 metabolism-related genes analyzed in *E. invadens*), comparison to our data will still be instructive, as genes or pathways identified as being differentially expressed by two different methods are highly likely to be truly developmentally regulated. However, due to the small number of observations in these studies, many regulated genes were likely missed. Comparison of our results to the cyst proteome showed no significant overlap; of the 195 proteins identified as cyst-specific, 74 had identifiable *E. invadens *orthologs, and only 14 of these were up-regulated in at least one encystation time point. Genes in this category included those involved in cyst wall synthesis, such as the chitin binding protein EIN_040990, alpha-amylase (EIN_052160) and a putative MADS-box transcription factor (EIN_161180). Whether this poor overlap was due to differences in cyst biology between the two species, misidentification of *E. invadens *orthologs, or reflects a difference between gene expression and protein levels is unclear. However, when our data were compared to the *E. invadens *microarray data [56], more similarities were identified, with 37 of the 89 genes down-regulated during encystation in the microarray experiment also significantly down-regulated by RNA-Seq (*P *≈ 9.7 × 10^-7^). Overlap between the up-regulated genes was not significant (14 out of the 51 total up-regulated genes), likely because the genes observed by Jeelani *et al. *[56] were limited to basic metabolic processes, a sample which our data shows was heavily down-regulated during encystation. Differences between these two datasets may also reflect differing basal expressions of genes in the trophozoite stage. Genes down-regulated in Jeelani *et al*. but not in our study generally had low basal expression levels based on the RNA-Seq data; hence, it was not surprising that these genes were not further down-regulated during encystation. The reverse pattern was seen in the up-regulated genes that did not overlap - relatively high basal expression levels - indicating that these genes were already expressed at sufficient levels prior to encystation. These differences in basal expression may be caused by changes to the IP-1 strain during passage in different laboratories or be due to media conditions (LYI-S-2 versus BI-S-33), which could affect expression of metabolic genes. Similar variation between laboratories has been noted in microarray studies of *E. histolytica *gene expression [57].

### Functions of developmentally regulated genes

To better understand the molecular processes underpinning development, we examined functional domains of regulated genes identified in the pairwise comparisons. Protein sequences for all genes up-regulated early in encystation (8 to 24 h encystation compared to trophozoites), genes up-regulated and down-regulated late in encystation (72 h encystation compared to trophozoites), and genes up-regulated during excystation (2 h excystation compared to mature, 72 h cysts) were chosen as likely to be the most biologically relevant. The majority of *E. invadens *genes encode hypothetical proteins of unknown function; hence, protein sequences for all significantly regulated genes were obtained from AmoebaDB [58] and searched for functional domains using the Pfam database [47]. Significantly enriched domains were identified by comparing to the frequency of each domain in the whole genome. Selected Pfam domains are shown in Table 4, and a complete list with all significant domains can be found in Additional file 10. To further enhance our understanding of the roles developmentally regulated genes may be playing in stage conversion, we also undertook an analysis of GO term [59] enrichment among significantly regulated genes. The top categories at each analyzed time point are listed in Table 5 and the complete results (all categories with enrichment *P*-values <0.05) are in Additional file 10.

**Table 4:** Pfam domains enriched in up- and down-regulated genes

Pfam domain ID^a^	Pfam domain description	Domains in genome^b^	Domains in subset^c^	*P*-value^d^
**Up-regulated at 8 h encystation (compared to trophozoite)**				
PF00962	Adenosine/AMP deaminase	10	4	1.00 × 10^-4^
PF01370	NAD dependent epimerase/dehydratase family	7	3	6.45 × 10^-4^
PF01380	Sugar isomerase domain	3	2	2.20 × 10^-3^
PF01416	tRNA pseudouridine synthase	5	2	6.92 × 10^-3^
PF06978	Ribonucleases P/MRP protein subunit POP1	2	2	7.52 × 10^-4^
PF02463	Structural maintenance of chromosomes superfamily	9	3	1.46 × 10^-3^
PF08221	RNA polymerase III subunit RPC82 helix-turn-helix domain	3	2	2.20 × 10^-3^
PF00620	RhoGAP domain	97	10	2.61 × 10^-4^
PF00566	Rab-GTPase-TBC domain	62	6	5.48 × 10^-3^
PF08743	DNA repair	3	2	2.20 × 10^-3^
PF03114	Protein dimerisation domain	12	3	3.53 × 10^-3^
PF09777	Osteopetrosis-associated transmembrane protein 1 precursor	2	2	7.52 × 10^-4^
PF00888	Cullin family ubiquitin ligase scaffold protein	11	3	2.72 × 10^-3^
PF10557	Cullin protein neddylation domain	12	3	3.53 × 10^-3^
PF00643	B-box zinc finger	15	3	6.72 × 10^-3^
PF00578	Alkyl hydroperoxide reductase and thiol specific antioxidant	17	3	9.50 × 10^-3^
PF12796	Ankyrin repeats	87	9	5.03 × 10^-4^
PF03197	Bacteriophage FRD2 protein	3	2	2.20 × 10^-3^
PF07534	unknown function	210	19	4.34 × 10^-6^
**Up-regulated at 24 h encystation (compared to trophozoite)**				
PF06045	Rhamnogalacturonate lyase family	6	5	2.74 × 10^-6^
PF00704	Glycosyl hydrolases family 18	7	5	9.07 × 10^-6^
PF07651	Phosphatidylinositol 4,5-bisphosphate binding	8	4	4.96 × 10^-4^
PF07653	Variant SH3 domain	21	6	6.16 × 10^-4^
PF00018	SH3 domain: protein-protein interaction	32	7	1.19 × 10^-3^
PF00614	Phospholipase D active site motif	2	2	2.98 × 10^-3^
PF00069	Protein kinase domain	510	41	3.42 × 10^-3^
PF00806	Pumilio-family RNA binding repeat	60	12	6.48 × 10^-5^
PF06978	Ribonucleases P/MRP protein subunit POP1	2	2	2.98 × 10^-3^
PF00352	Transcription factor TFIID (or TATA-binding protein, TBP)	11	4	1.98 × 10^-3^
PF00620	RhoGAP domain	97	18	3.50 × 10^-6^
PF00225	Kinesin motor domain	6	3	2.75 × 10^-3^
PF03953	Tubulin C-terminal domain	6	3	2.75 × 10^-3^
PF00443	Ubiquitin carboxyl-terminal hydrolase	29	6	3.45 × 10^-3^
PF13476	ATPase domain	10	4	1.33 × 10^-3^
PF04506	Integral membrane protein, possible sugar transporter	2	2	2.98 × 10^-3^
PF12796	Ankyrin repeats	87	13	6.03 × 10^-4^
PF07534	Unknown function	210	31	3.07 × 10^-7^
**Up-regulated at 72 h encystation (compared to trophozoite)**				
PF00128	Alpha amylase, catalytic domain	24	7	5.95 × 10^-5^
PF02784	Pyridoxal-dependent decarboxylase, pyridoxal binding domain	5	3	8.37 × 10^-4^
PF12697	Alpha/beta hydrolase family	8	3	4.08 × 10^-3^
PF00614	Phospholipase D active site motif	2	2	2.04 × 10^-3^
PF00806	Pumilio-family RNA binding repeat	60	12	1.05 × 10^-5^
PF00773	Catalytic domain of ribonuclease II	10	4	6.58 × 10^-4^
PF01885	RNA 2'-phosphotransferase, Tpt1/KptA family	3	2	5.83 × 10^-3^
PF06978	Ribonucleases P/MRP protein subunit POP1	2	2	2.04 × 10^-3^
PF04054	CCR4-Not complex component - global regulator of transcription	5	3	8.37 × 10^-4^
PF00145	C-5 cytosine-specific DNA methylase	2	2	2.04 × 10^-3^
PF08221	RNA polymerase III subunit RPC82 helix-turn-helix domain	3	2	5.83 × 10^-3^
PF08743	DNA repair	3	2	5.83 × 10^-3^
PF02144	Repair protein Rad1/Rec1/Rad17	3	2	5.83 × 10^-3^
PF02145	Rap/ran-GAP	33	6	2.67 × 10^-3^
PF00620	RhoGAP domain	97	11	2.92 × 10^-3^
PF04506	Integral membrane protein, possible sugar transporter	2	2	2.04 × 10^-3^
PF01436	NHL repeat	8	3	4.08 × 10^-3^
PF05237	MoeZ/MoeB domain: unknown function	3	2	5.83 × 10^-3^
PF07534	Unknown function	210	20	8.17 × 10^-4^
**Up-regulated at 2 h excystation (compared to 72 h cyst)**				
PF00723	Glycosyl hydrolases family 15	6	4	4.71 × 10^-4^
PF00534	Glycosyl transferases group 1	4	3	1.75 × 10^-3^
PF01592	Iron-sulfur cluster assembly	148	24	2.86 × 10^-4^
PF00076	RNA recognition motif (aka RRM, RBD, or RNP domain)	36	9	1.11 × 10^-3^
PF00270	DEAD/DEAH box helicase	12	5	1.29 × 10^-3^
PF00565	Staphylococcal nuclease homolog	5	3	4.03 × 10^-3^
PF01416	Pseudouridine synthesis	4	3	1.75 × 10^-3^
PF04719	hTAFII28-like protein conserved region	9	4	3.10 × 10^-3^
PF01926	50S ribosome-binding GTPase	2	2	6.08 × 10^-3^
PF02270	Transcription initiation factor IIF (TFIIF)	22	7	8.81 × 10^-4^
PF00226	DnaJ domain	4	3	1.75 × 10^-3^
PF08423	DNA repair and recombination	19	6	2.12 × 10^-3^
PF02450	Lecithin:cholesterol acyltransferase	2	2	6.08 × 10^-3^
PF06728	GPI transamidase subunit	2	2	6.08 × 10^-3^
PF01238	Phosphomannose isomerase type I	46	10	1.81 × 10^-3^
PF00112	Papain family cysteine protease	6	3	7.44 × 10^-3^
PF01067	Calpain large subunit, domain III (protease)	28	20	1.03 × 10^-16^
PF00630	Filamin/ABP280 repeat	24	9	4.09 × 10^-5^
PF01602	Adaptin N-terminal region	9	6	1.47 × 10^-5^
PF04324	BFD-like [2Fe-2S] binding domain	4	3	1.75 × 10^-3^
PF02777	Iron/manganese superoxide dismutases, C-terminal domain	8	4	1.87 × 10^-3^
PF00400	WD domain, G-beta repeat	263	51	7.47 × 10^-10^
PF13115	YtkA-like: unknown function	12	7	9.20 × 10^-6^
PF06229	FRG1-like family: unknown function	4	3	1.75 × 10^-3^
PF08238	Sel1 repeat	21	6	3.60 × 10^-3^
**Down-regulated at 72 h encystation (compared to trophozoite)**				
PF00723	Glycosyl hydrolases family 15	6	4	5.42 × 10^-4^
PF00106	Short chain dehydrogenase	12	6	1.55 × 10^-4^
PF01663	Type I phosphodiesterase/nucleotide pyrophosphatase	19	7	4.13 × 10^-4^
PF02878	Phosphoglucomutase/phosphomannomutase domain I	3	3	5.29 × 10^-4^
PF02880	Phosphoglucomutase/phosphomannomutase domain III	3	3	5.29 × 10^-4^
PF00408	Phosphoglucomutase/phosphomannomutase, C-terminal domain	3	3	5.29 × 10^-4^
PF00349	Phosphorylates hexoses	2	2	6.54 × 10^-3^
PF01467	Cytidylyltransferase	9	6	1.82 × 10^-5^
PF09334	tRNA synthetases class I (M)	3	3	5.29 × 10^-4^
PF00432	Prenyltransferase and squalene oxidase repeat	4	4	4.27 × 10^-5^
PF00630	Filamin/ABP280 repeat	28	25	1.10 × 10^-24^
PF00307	Calponin homology (CH) domain	50	13	9.75 × 10^-5^
PF06268	Actin cross-linking	6	5	1.90 × 10^-5^
PF01602	Adaptin N-terminal region	24	9	5.41 × 10^-5^
PF04324	BFD-like [2Fe-2S] binding domain	9	6	1.82 × 10^-5^
PF00248	Aldo/keto reductase family	4	4	4.27 × 10^-5^
PF02777	Iron/manganese superoxide dismutases, C-terminal domain	4	4	4.27 × 10^-5^
PF00081	Iron/manganese superoxide dismutases, alpha-hairpin domain	5	4	1.96 × 10^-4^
PF13115	YtkA-like: unknown function	12	8	6.40 × 10^-7^
PF00501	AMP-binding enzyme	26	11	2.08 × 10^-6^
PF12796	Ankyrin repeats	87	20	1.15 × 10^-5^

^a^Selected Pfam domains that were significantly enriched (*P *<0.05) in genes up- or down-regulated at 8 h, 24 h and 72 h post-encystation, and at 2 h post-excystation. ^b^Number of a Pfam domain in the whole *E. invadens *genome. ^c^Number of a Pfam domain among the subset of regulated genes. ^d^*P*-value for Pfam domain enrichment.

**Table 5:** Gene Ontology terms enriched in up- and down-regulated genes

GO ID^a^	GO category name	Genes in genome^b^	Genes in subset^c^	*P*-value^d^
**Up-regulated at 8 h encystation (compared to trophozoite)**				
GO:0006030	Chitin metabolism	5	5	1.21 × 10^-7^
GO:0006041	Glucosamine metabolism	5	5	1.21 × 10^-7^
GO:0006807	Nitrogen compound metabolism	33	7	3.33 × 10^-4^
GO:0009308	Amine metabolism	27	6	6.94 × 10^-4^
GO:0005975	Carbohydrate metabolism	115	11	7.92 × 10^-3^
GO:0001522	Pseudouridine synthesis	7	3	2.23 × 10^-3^
GO:0007017	Microtubule-based process	13	4	1.57 × 10^-3^
**Up-regulated at 24 h encystation (compared to trophozoite)**				
GO:0006030	Chitin metabolism	5	3	4.10 × 10^-3^
GO:0006807	Nitrogen compound metabolism	33	7	1.15 × 10^-2^
GO:0009308	Amine metabolism	27	6	1.52 × 10^-2^
GO:0016311	Dephosphorylation	54	9	2.13 × 10^-2^
GO:0006351	Transcription, DNA-dependent	35	6	4.96 × 10^-2^
GO:0006367	Transcription initiation from RNA polymerase II promoter	11	3	4.78 × 10^-2^
GO:0007017	Microtubule-based process	13	6	2.22 × 10^-4^
GO:0007018	Microtubule-based movement	6	3	7.74 × 10^-3^
**Up-regulated at 72 h encystation (compared to trophozoite)**				
GO:0009966	Regulation of signal transduction	120	13	7.62 × 10^-3^
GO:0007275	Development	10	3	1.22 × 10^-2^
GO:0040029	Regulation of gene expression, epigenetic	8	3	6.14 × 10^-3^
GO:0006139	Nucleobase, nucleoside, nucleotide and nucleic acid metabolism	328	24	4.39 × 10^-2^
GO:0051056	Regulation of small GTPase mediated signal transduction	120	13	7.62 × 10^-3^
GO:0007017	Microtubule-based process	13	3	2.59 × 10^-2^
**Up-regulated at 2 h excystation (compared to 72 h cyst)**				
GO:0019318	Hexose metabolism	21	8	9.38 × 10^-4^
GO:0005975	Carbohydrate metabolism	115	23	2.00 × 10^-3^
GO:0044262	Cellular carbohydrate metabolism	35	10	2.79 × 10^-3^
GO:0006096	Glycolysis	7	4	3.51 × 10^-3^
GO:0006007	Glucose catabolism	8	4	6.42 × 10^-3^
GO:0006006	Glucose metabolism	17	6	6.47 × 10^-3^
GO:0006066	Alcohol metabolism	28	8	7.31 × 10^-3^
GO:0051186	Cofactor metabolism	54	12	1.02 × 10^-2^
GO:0016052	Carbohydrate catabolism	10	4	1.62 × 10^-2^
GO:0016226	Iron-sulfur cluster assembly	7	4	3.51 × 10^-2^
GO:0042254	Ribosome biogenesis and assembly	18	5	3.65 × 10^-2^
GO:0008654	Phospholipid biosynthesis	14	5	1.22 × 10^-2^
GO:0046467	Membrane lipid biosynthesis	14	5	1.22 × 10^-2^
GO:0006644	Phospholipid metabolism	25	7	1.34 × 10^-2^
GO:0006497	Protein lipidation	10	4	1.62 × 10^-2^
GO:0006508	Proteolysis	138	25	5.27 × 10^-3^
GO:0006996	Organelle organization and biogenesis	112	21	6.85 × 10^-3^
GO:0006800	Oxygen and reactive oxygen species metabolism	5	3	1.04 × 10^-2^
**Up-regulated at 8 h excystation (compared to 72 h cyst)**				
GO:0006066	Alcohol metabolism	28	8	1.26 × 10^-2^
GO:0005975	Carbohydrate metabolism	115	22	1.30 × 10^-2^
GO:0019752	Carboxylic acid metabolism	26	8	7.82 × 10^-3^
GO:0009059	Macromolecule biosynthesis	202	34	1.72 × 10^-2^
GO:0006260	DNA replication	56	12	2.65 × 10^-2^
GO:0006139	Nucleobase, nucleoside, nucleotide and nucleic acid metabolism	328	50	2.69 × 10^-2^
GO:0006259	DNA metabolism	144	25	2.71 × 10^-2^
GO:0016568	Chromatin modification	11	4	3.17 × 10^-2^
GO:0006629	Lipid metabolism	71	19	3.61 × 10^-4^
GO:0008610	Lipid biosynthesis	21	7	7.88 × 10^-3^
GO:0044255	Cellular lipid metabolism	38	10	1.02 × 10^-2^
GO:0008654	Phospholipid biosynthesis	14	5	1.78 × 10^-2^
GO:0006644	Phospholipid metabolism	25	7	2.15 × 10^-2^
GO:0006497	Protein lipidation	10	4	2.22 × 10^-2^
GO:0006508	Proteolysis	138	25	1.65 × 10^-2^
GO:0006996	Organelle organization and biogenesis	112	21	1.87 × 10^-2^
**Down-regulated at 8 h encystation (compared to trophozoite)**				
GO:0009309	Amine biosynthesis	10	3	1.38 × 10^-3^
GO:0006519	Amino acid and derivative metabolism	23	4	1.86 × 10^-3^
GO:0005975	Carbohydrate metabolism	115	8	5.37 × 10^-3^
GO:0019752	Carboxylic acid metabolism	26	3	2.27 × 10^-2^
GO:0051186	Cofactor metabolism	54	4	3.81 × 10^-2^
GO:0016226	Iron-sulfur cluster assembly	7	4	9.80 × 10^-6^
GO:0050896	Response to stimulus	46	4	3.14 × 10^-2^
GO:0006323	DNA packaging	39	4	1.81 × 10^-2^
GO:0051276	Chromosome organization and biogenesis	43	4	2.52 × 10^-2^
GO:0006259	DNA metabolism	144	8	3.36 × 10^-2^
GO:0046467	Membrane lipid biosynthesis	14	3	3.90 × 10^-3^
GO:0006644	Phospholipid metabolism	25	3	2.04 × 10^-2^
GO:0006497	Protein lipidation	10	3	1.38 × 10^-3^
**Down-regulated at 24 h encystation (compared to trophozoite)**				
GO:0005975	Carbohydrate metabolism	115	7	2.99 × 10^-2^
GO:0050896	Response to stimulus	46	4	3.14 × 10^-2^
GO:0006323	DNA packaging	39	4	1.81 × 10^-2^
GO:0051276	Chromosome organization and biogenesis	43	4	2.52 × 10^-2^
GO:0006259	DNA metabolism	144	8	3.36 × 10^-2^
GO:0031497	Chromatin assembly	30	3	4.27 × 10^-2^
GO:0006461	Protein complex assembly	31	3	4.64 × 10^-2^
**Down-regulated at 72 h encystation (compared to trophozoite)**				
GO:0006096	Glycolysis	7	5	2.22 × 10^-4^
GO:0019320	Hexose catabolism	8	5	5.42 × 10^-4^
GO:0046365	Monosaccharide catabolism	8	5	5.42 × 10^-4^
GO:0006007	Glucose catabolism	8	5	5.42 × 10^-4^
GO:0005996	Monosaccharide metabolism	22	8	1.19 × 10^-3^
GO:0005975	Carbohydrate metabolism	115	23	1.58 × 10^-3^
GO:0006092	Main pathways of carbohydrate metabolism	12	5	5.35 × 10^-3^
GO:0016052	Carbohydrate catabolism	10	5	2.04 × 10^-3^
GO:0016226	Iron-sulfur cluster assembly	7	4	3.28 × 10^-3^
GO:0031163	Metallo-sulfur cluster assembly	7	4	3.28 × 10^-3^
GO:0006629	Lipid metabolism	71	15	5.95 × 10^-3^
GO:0051258	Protein polymerization	4	3	4.29 × 10^-3^
GO:0030036	Actin cytoskeleton organization and biogenesis	7	4	3.28 × 10^-3^
GO:0030041	Actin filament polymerization	4	3	4.29 × 10^-3^
GO:0008154	Actin polymerization and/or depolymerization	4	3	4.29 × 10^-3^
GO:0016043	Cell organization and biogenesis	306	51	3.70 × 10^-4^
GO:0006996	Organelle organization and biogenesis	112	21	5.58 × 10^-3^
GO:0006800	Oxygen and reactive oxygen species metabolism	5	4	5.58 × 10^-4^
GO:0006801	Superoxide metabolism	5	4	5.58 × 10^-4^
**Down-regulated at 2 h excystation (compared to 72 h cyst)**				
GO:0007154	Cell communication	566	23	3.73 × 10^-2^
GO:0016043	Cell organization and biogenesis	306	14	4.47 × 10^-2^
**Down-regulated at 8 h excystation (compared to 72 h cyst)**				
GO:0008643	Carbohydrate transport	5	3	3.56 × 10^-3^
GO:0044249	Cellular biosynthesis	276	51	1.18 × 10^-10^
GO:0006412	Protein biosynthesis	194	44	2.14 × 10^-12^
GO:0006414	Translational elongation	11	3	4.21 × 10^-2^
GO:0051276	Chromosome organization and biogenesis	43	9	3.42 × 10^-3^
GO:0006323	DNA packaging	39	8	6.49 × 10^-3^
GO:0031497	Chromatin assembly	30	6	2.01 × 10^-2^
GO:0006334	Nucleosome assembly	23	6	5.27 × 10^-3^
GO:0006461	Protein complex assembly	31	7	6.20 × 10^-3^
GO:0016043	Cell organization and biogenesis	306	32	2.40 × 10^-2^
GO:0006996	Organelle organization and biogenesis	112	14	3.41 × 10^-2^

^a^Selected GO terms that were significantly enriched (*P *< 0.05) in genes up- or down-regulated at 8 h, 24 h and 72 h post-encystation, and at 2 h post-excystation. ^b^Number of genes in a GO category in the whole *E. invadens *genome. ^c^Number of genes in a GO category among the subset of regulated genes. ^d^*P*-value for GO category enrichment.

#### Early encystation

Numerous gene families involved in signal transduction were significantly up-regulated early in encystation, including signaling molecules such as protein kinases, small GTPase activating proteins, and lipid signaling proteins. Similar results were seen in *E. histolytica *cysts, where numerous kinases and other potential signaling pathway members were observed to be up-regulated in cysts [9]. These proteins may be involved in transducing and affecting the signals that trigger encystation. Previous studies using small molecule agonists and inhibitors have suggested pathways that may help trigger stage conversion. Catecholamines, which in vertebrate cells stimulate signaling through the β-adrenergic receptor, were found to stimulate encystation in *E. invadens *trophozoites [60]. Interestingly, PLD, which has been found to transduce signals from a receptor in rat cortical astrocytes [61], is strongly up-regulated early in encystation, as well as other potential modulators of G-protein coupled receptor signaling, such as small GTPase activating proteins and phosphatidylinositol 3-kinase (EIN_083000). Regulation of gene expression, whether at the transcriptional or post-transcriptional level, is crucial for stage conversion in many parasite species [62-64]. We found that Pfam domains associated with transcriptional regulation, such as helix-turn-helix motif DNA binding proteins and basal transcription factors such as the TATA binding protein, were highly enriched in genes up-regulated in early encystation. Multiple Myb family domain protein genes are regulated during development, including one (EIN_241120) that is highly homologous to the SHAQKY domain protein identified in *E. histolytica *(originally identified in [29] as EIN_052670). These transcription factors may drive cell fate decisions during encystation by promoting expression of cyst-specific genes.

Interestingly, RNA metabolism was also regulated during encystation, with RNA binding proteins, RNAseP domain proteins, and the RNA-editing protein pseudouridine synthase (EIN_156980) up-regulated in early cysts. This last gene is particularly interesting, as in the apicomplexan parasite *Toxoplasma gondii *mutations in PUS1, an RNA pseudouridine synthase, were found to sharply decrease rates of differentiation from the tachyzoite to bradyzoite forms [65]; it is possible that a similar dependence on RNA editing is found in *Entamoeba *development. In observing the enriched GO terms, we found, as expected, that genes involved in glucosamine metabolism (GO:0006041), important for cyst wall synthesis, are up-regulated early in encystation. Additional GO terms enriched among early up-regulated genes include microtubule based processes (GO:0007017) and DNA dependent transcription (GO:0006351).

#### Late encystation

Many down-regulated genes in mature cysts encode proteins involved in basic metabolic processes, such as phosphoglucomutase (EIN_242460), hexokinases (EIN_040150, EIN_015200) and short-chain dehydrogenases (EIN_315930, EIN_147830, EIN_239670, EIN_222000, EIN_039970, EIN_243560). This finding is consistent with recent work [56] in which the metabolome of encysting *E. invadens *was determined. In this study it was observed that during encystation and in mature cysts (48 h and 120 h post-encystation), basic metabolic processes such as glycolysis were drastically decreased, and glucose metabolism redirected to cyst wall synthesis. Similar to the findings in *E. histolytica *[9], numerous virulence factors were also down-regulated in mature cysts, which may be expected as this stage does not cause disease symptoms in the host. In addition to constituents of the Gal/GalNac lectin complex previously mentioned, genes of the serine, threonine, isoleucine rich proteins (STIRP) (EIN_110790), rhomboid protease (EIN_219350, EIN_255710) and peroxiredoxin (EIN_174460, EIN_130760, EIN_004350 and EIN_076200) families, which have demonstrated virulence activities in *E. histolytica *[66-68], have decreased expression in mature cysts compared to trophozoites.

RNA metabolism continues to be regulated in mature cysts, with multiple Pumilio homology domain proteins (EIN_033210, EIN_156840) and the ribonucleoprotein EIN_004530 being up-regulated. These proteins may be involved in formation of the chromatoid bodies, RNP structures that are found in *Entamoeba *cysts [69]. In addition, DNA repair pathway genes such as the Rad1 homolog EIN_013450 and the Rad52 homolog EIN_094590 have increased expression and may facilitate nuclear division, which occurs late in encystation [70]. Interestingly, DNA repair genes were previously observed to be a significantly enriched group among genes up-regulated in *E. histolytica *cysts [9], indicating that they may be involved in a process common to encystation in all *Entamoeba *species. Consistent with recent findings that levels of most amino acids decrease in encystation [56], genes involved in amino acid metabolism (GO:0006519) are down-regulated. Later in encystation, chromatin assembly (GO:0006333) and DNA metabolism (GO:0006139) genes are up-regulated. As with the DNA repair-related genes noted earlier, genes in these groups may be important for nuclear division. Consistent with our Pfam family analysis, carbohydrate metabolism (GO:0005975) was significantly enriched in genes down-regulated at 48 and 72 h of encystation. In addition, other metabolic pathways, including lipid metabolism (GO:0006629) and biosynthesis (GO:0009058), are reduced in mature cysts.

#### Excystation

The down-regulation of carbohydrate metabolism observed in mature cysts is reversed during excystation, with increased transcript levels of glycoside hydrolases (EIN_135910, EIN_106440) as well as the hexokinases that had been down-regulated during encystation. Other gene families up-regulated during excystation include likely regulators of transcription, such as TFIID (EIN_111320), and protein synthesis, such as tRNA synthetases (EIN_222960, EIN_156340) and a PIG-U that is involved in GPI-anchor synthesis (EIN_019990). Regulation of these genes is consistent with synthesis of proteins required for trophozoite function. Our finding that cysteine proteases are significantly up-regulated during excystation is consistent with data showing that cysteine protease inhibitors inhibit excystation [71], and may indicate a role for these proteases in degrading the cyst wall. GO analysis showed that glycolytic pathways (GO:0006096), lipid biosynthesis (GO:0008610) and ribosome assembly (GO:0042254) genes show increased expression in excysting parasites.

### Meiosis-specific genes are upregulated during encystation

In common with many protozoa for which no sexually dimorphic forms could be identified (for example, kinetoplastids, *Giardia*, trichomonads), the *Entamoebae *were long thought to be asexual. However, many of these protozoa show evidence of sexuality (meiotic cell division and genetic exchange) [72-74]. Comparative analysis of many eukaryotic species has shown that *E. histolytica *contains most of the machinery required for meiosis [73], and our orthology analysis identified these genes in *E. invadens*. Additionally, a previous analysis of *E. histolytica *genomes demonstrated haplotype structures that strongly suggest sexual recombination [75]. However, how and when recombination occurs is not known. Nuclear division occurs during encystation as trophozoites have one nucleus while cysts (of *E. invadens *and *E. histolytica*, though not all *Entamoeba *species) have four [70,76]. We hypothesize that meiosis occurs during encystation, with the two divisions (meiosis I and meiosis II) resulting in four haploid nuclei.

**Figure 8:**
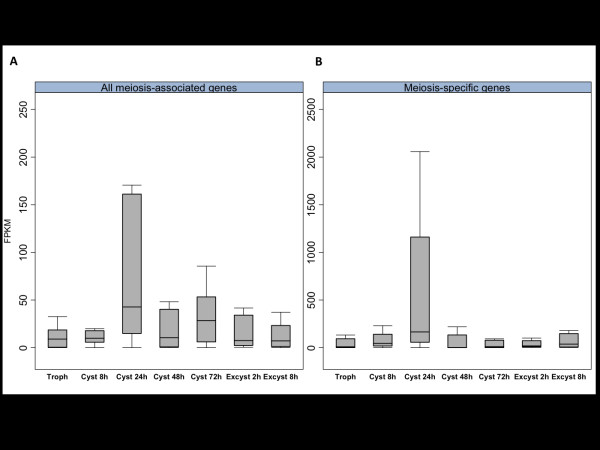
**Changes in expression levels of genes involved in meiosis**. **(a,b) **Boxplots showing the distribution of expression values at each time point during encystation and excystation for meiosis-associated but not meiosis-specific genes (a) and for genes with meiosis-specific roles (b). There is a trend towards increased expression at 24 h after induction of encystation. This pattern of expression is consistent with a model in which meiotic division gives rise to the four nuclei in the cyst.

We analyzed the expression patterns of meiosis-specific genes (those genes that are only involved in meiosis) and all meiosis genes (including genes that are also involved in other nuclear maintenance processes, including mitosis) [73]. Figure 8 shows the median and distribution of expression values of all genes in these groups; Additional file 11 gives the FPKM for each gene. The data demonstrate clear up-regulation of expression in all meiosis-associated and meiosis-specific genes at 24 hours after the induction of encystation. Meiosis-specific MND1 (EIN_051380) and HOP2 (EIN_249340) form a complex to bind to DNA at double strand breaks [77]. They are both very strongly up-regulated in our data with the highest FPKM values of all the meiosis genes at 8 h and 24 h of encystation. MND1, which stabilizes the heteroduplex after double strand break formation is up-regulated four-fold at 24 h of encystation. DMC1 (EIN_249340), a meiosis homolog of RAD52 [78], which promotes recombination between homologs, is massively up-regulated at 24 h (FPKM = 3.7 at 0 h and 263.6 at 24 h of encystation) before returning to low level expression at 72 h (FPKM = 0.8). Its mitotic homolog RAD52 (EIN_094590) remains up-regulated after 24 h. MSH4 and MSH5 (EIN_222600, EIN_020760) are meiosis-specific and form a heterodimer involved in Holliday junction resolution [79]; the *MSH4 *gene has very low levels of transcription and is detected only at 8 h during encystation whereas *MSH5 *shows peak levels at 24 h. Global analysis of the meiosis-associated but non-specific genes also shows a clear pattern of up-regulation at approximately 24 h during encystation. This is consistent with the data on meiosis-specific genes and supports our hypothesis that meiosis is occurring during cyst formation.

Meiosis during encystation is consistent with cysts being a dispersal stage for the parasite. Genetic exchange and expression of meiosis-specific genes has also been described in *Giardia *cysts, although the process involved may be non-meiotic [74]. During dispersal, it could be advantageous for the parasite to recombine, as this may enable it to infect more diverse hosts. In *Entamoeba *it is not yet proven that recombination occurs, but if the nuclei in the cysts are haploid, then there must be some form of nuclear fusion during excystation in order to produce diploid trophozoites.

### Phospholipase D is required for efficient encystation in *E. invadens*

**Figure 9:**
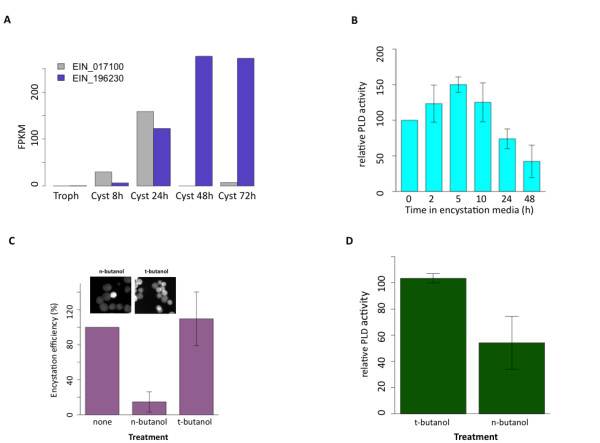
**Phospholipase D expression and function during encystation**. The potential role of PLD in encystation was examined by observing the changes in expression and enzyme activity, and the effect of inhibition of PLD on encystation efficiency. **(a) **Expression of two *E. invadens *PLD genes. FPKM values for EIN_017100 and EIN_196230 at each encystation time point are shown. **(b) **PLD enzyme activity was measured using the Amplex Red Phospholipase D kit (Molecular Probes). Relative activity (measured as fluorescence at 585 nm and normalized to trophozoite activity) is shown for each time point. Error bars indicate ± standard deviation. **(c) **Inhibition of PLD decreases encystation efficiency. Encysting cultures of *E. invadens *were either untreated, treated with 0.6% n-butanol (an inhibitor of PLD activity) or with 0.6% t-butanol (which has no effect on PLD activity). Significant reduction of encystation efficiency (*P *< 0.01) was seen with n-butanol treatment, when compared to untreated parasites, but efficiency did not change with addition of t-butanol. Error bars indicate ± standard deviation. Insets show representative images of n-butanol and t-butanol treated samples, stained with calcofluor, which labels cysts. Note the higher percentage of positively staining cells in the t-butanol treated sample. **(d) **Inhibition of *E. invadens *PLD by n-butanol. To confirm that the effect of n-butanol on encystation was due to inhibition of PLD, enzyme activity of trophozoite lysate was measured in the presence of either n- or t-butanol. Relative activity (measured as fluorescence at 585 nm and normalized to activity in untreated lysate) is shown for each condition. Error bars indicate ± standard deviation.

Among the genes with increasing expression during encystation was that encoding PLD, an enzyme involved in lipid second messenger signaling. PLD catalyzes the conversion of phosphatidyl choline to phosphatidic acid and has been linked to many important biological processes, including vesicle transport and transduction of signals required for cell shape changes and proliferation [80,81]. *E. invadens *has two genes encoding PLDs: EIN_017100 and EIN_196230. Both are highly up-regulated during encystation (Figure 9a). PLD was also up-regulated in *E. histolytica *cysts [9].

To determine if there was a regulatory role for PLD in encystation, we undertook functional studies. First, we examined changes in PLD activity during development. Using the Amplex Red Phospholipase D Assay kit (Molecular Probes), we assayed PLD activity in whole cell lysates at 2 h, 5 h, 10 h, 24 h and 48 h after transfer to encystation media (Figure 9b). We found that PLD activity increased during encystation, peaking early (5 h) and falling back below trophozoite levels later in encystation (24 to 48 h). This pattern of activity supports a role for PLD during encystation; however, it does not coincide with peak RNA levels of the PLD genes determined by RNA-Seq, likely indicating that PLD activity is being regulated at the protein level. It should be noted that the activity assay cannot distinguish between the products of the two PLD genes; hence, differing activity levels for the two enzymes could further complicate the relationship between activity and gene expression.

We then tested whether inhibition of PLD activity affected encystation efficiency. PLD inhibition was achieved by addition of 0.6% n-butanol, which acts as a non-productive substrate for PLD, suppressing formation of phosphatidic acid [82]; the same amount of tert-butanol, which has no effect on PLD activity, was used as a control. N-butanol was added to encystation media upon introduction of encystation in trophozoites; encystation was allowed to proceed for 48 h, after which encystation efficiency was assayed by treatment with 0.1% sarkosyl. We found a marked reduction of encystation efficiency (approximately 22% of untreated levels, *P *< 0.01) in the n-butanol treated samples (Figure 9c); however, cysts that formed in n-butanol treated cultures were normal in size and gross morphology. Addition of t-butanol had no significant effect on encystation, confirming the specificity of the n-butanol repression of encystation. To ensure that this effect was indeed due to inhibition of PLD by n-butanol, we tested susceptibility of the *E. invadens *PLD to butanol using the activity assay described above. We found that addition of 0.6% n-butanol to the reaction mixture significantly reduced PLD activity, while no effect was seen with the same amount of t-butanol (Figure 9d). These results indicate that PLD could be an important regulator of encystation in *Entamoeba*. Whether PLD is required for transduction of the initial signals that trigger encystation, perhaps via a G-protein coupled receptor, or is a downstream effector will require further study.

PLD has been implicated in cell fate regulation and other developmental processes in a wide range of species, including zoospore differentiation in the fungus *Phytophthora infestans *[83], quorum sensing in *Dictyostelium *[84] and regulation of proliferation in mammalian systems [61,80], where extensive crosstalk between PLD signaling and other critical pathways such as sphingolipid signaling and protein kinase C has been documented [85,86]. In addition to PLD, other potential regulators of lipid signaling and protein kinase C activity are up-regulated during encystation, including diacyglycerol kinase (EIN_094160), phosphoinositol 3-kinase (EIN_083000) and a homolog of ceramide synthase (EIN_255100), potentially indicating a role for these pathways in encystation. Further investigation will be required to determine if PLD and protein kinase C pathways interdigitate in *Entamoeba *as they do in other systems, and to identify how they contribute to the signaling network controlling development. The identification of a regulator of encystation by finding genes with differential expression by RNA-Seq suggests that this data set will be an important source of information about *Entamoeba *development, and provide many targets for future inquiry, including potential genes to target for inhibition of stage conversion.

## Conclusions

Encystation and excystation are vital for dispersal and pathogenicity in some of the most important intestinal pathogens affecting humans, including *Giardia*, *Cryptosporidium *and *Entamoeba*, and their potential as targets for therapeutic intervention has recently been highlighted [87]. However, encysting organisms can be very distantly related and it is unlikely that they have conserved many of the mechanistic features of the process over these long evolutionary periods; rather, these similarities may represent convergent adaptation to analogous lifestyles and environments. By understanding the similarities between these processes, we can begin to understand common selective forces acting on these parasites and potentially common therapeutic targets. The genomic and transcriptomic data described in this paper will lay the foundation for functional studies of the developmental cycle in *Entamoeba*. Our study has shown a number of important similarities between the processes in *Giardia *and *Entamoeba*, including down-regulation of basic metabolic processes [88,89], meiotic division, and involvement of Myb domain transcription factors and lipid signaling pathways. We have also described potential signaling mechanisms that could be involved in triggering the encystation process. These genome-wide datasets lay the groundwork for future mechanistic dissection of the developmental cascade and identification of new targets for diagnostic or treatment approaches.

## Materials and methods

### *E. invadens *genome assembly and gene prediction

The sequenced strain of *E. invadens*, IP-1, was originally isolated from a natural infection of a painted turtle, *C. picta*, and was pathogenic in snakes [5]. The genome was sequenced at the J Craig Venter Institute (JCVI) sequencing center. Genomic DNA was sheared by sonication and cloned into pHOS2 plasmid vectors to generate small (1.5 to 2 kb) and medium (4 to 5 kb) insert libraries, which were sequenced using dye terminator sequencing on ABI 3730 sequencers, generating 294,620 reads. Reads were trimmed with UMD Overlapper [90] to determine a clear range for every read. Those with >98% BLASTN identity to the rRNA sequence of *E. invadens *were removed prior to genome assembly, as were tRNA sequences identified by tRNAscan-SE [91]. The remaining reads were assembled with Celera Assembler version 3.10 [23]. The following non-standard assembly options were used: the meryl K-mer frequency limit was set to 1,000 (default = 100) to allow more repetitive regions to seed overlaps; the assumed error rate for building unitigs was set to 0.5% (default = 1.5%) to separate similar repeats; the genome size (default = none) was set to 10 Mbp to reduce sensitivity to coverage-based repeat detection. The assembly ran on AMD Opteron processors with 64 GB RAM and the Suse 10.1 Linux operating system.

Generation of gene models for *E. invadens *was performed using a combination of *de novo *gene finders and homology based methods, utilizing the *E. histolytica *proteome as a reference. GeneZilla [92], Augustus [93] and Twinscan [94] were trained on a set of 500 manually curated gene models annotated using *E. histolytica *protein alignments. Protein alignments were performed with the Analysis and Annotation Tool (AAT) [95]. A final gene set was obtained using EVM, a consensus-based evidence modeler developed at JCVI [96].

The final consensus gene set was functionally annotated using the following programs: PRIAM [97] for enzyme commission (EC) number assignment, hidden Markov model (HMM) searches using Pfam [98] and TIGRfam [99] to discover conserved protein domains, BLASTP against JCVI internal non-identical protein database for protein similarity, SignalP for signal peptide prediction, TargetP to determine protein final destination, TMHMM for transmembrane domain prediction [100], and Pfam2go to transfer GO terms from Pfam hits that have been curated. An illustration of the JCVI Eukaryotic Annotation Pipeline (JEAP) components is shown in Additional file 1. All evidence was evaluated and ranked according to a priority rules hierarchy to give a final functional assignment reflected in a product name.

In addition to the above analyses, we performed protein clustering within the predicted proteome using a domain-based approach [101]. With this approach, proteins are organized into protein families to facilitate functional annotation, visualizing relationships between proteins and to allow annotation by assessment of related genes as a group, and rapidly identify genes of interest. This clustering method produces groups of proteins sharing protein domains conserved across the proteome, and consequently, related biochemical function.

For functional annotation curation we used Manatee [102]. Predicted *E. invadens *proteins were grouped on the basis of shared Pfam/TIGRfam domains and potential novel domains. To identify known and novel domains in *E. invadens*, the proteome was searched against Pfam and TIGRfam HMM profiles using HMMER3 [103]. For new domains, all sequences with known domain hits above the domain trusted cutoff were removed from the predicted protein sequences and the remaining peptide sequences were subject to all versus all BLASTP searches and subsequent clustering. Clustering of similar peptide sequences was done by linkage between any two peptide sequences having at least 30% identity over a minimum span of 50 amino acids, and an e-value <0.001. The Jaccard coefficient of community Ja,b was calculated for each linked pair of peptide sequences a and b, as follows: Ja,b = [(Number of distinct accessions matching a and b, including (a,b))/(Number of distinct accessions matching either a or b)]. The Jaccard coefficient Ja,b (also called the link score) represents the similarity between the two peptides a and b. The associations between peptides with a link score above 0.6 were used to generate single linkage clusters and aligned using ClustalW [104] and then used to develop conserved protein domains not present in the Pfam and TIGRfam databases. Any *E. invadens*-specific domain alignments containing five or more members were considered true domains for the purpose of clustering protein families. The peptides in the alignments were searched back against the *E. invadens *proteome to find additional members that may have been excluded during earlier stages due to the parameters employed. Full-length protein sequences were then grouped on the basis of the presence of Pfam/TIGRfam domains and potential novel domains. Proteins with exactly the same domain composition were then classified into putative domain-based protein families. All genome sequence and annotations have been deposited in GenBank under the Whole Genome Shotgun Assembly accession number [AANW00000000], Bioproject accession PRJNA12926 ID: 12926. Latest GenBank Assembly (EIA2v2) ID is GCA_000168215.2.

### *In vitro *culture of *E. invadens *and induction of stage conversion

*E. invadens *strain IP-1 [5] was maintained in LYI-S-2 [105] at 25°C. Encystation was induced by incubation in 47% LYI-LG, similar to previous methods [42], for 8 h, 24 h, 48 h or 72 h. For excystation, 72 h cysts were pre-incubated overnight (approximately 16 h) in distilled water at 4°C to lyse trophozoites, then induced to excyst by incubation in LYI-LG with the 1 mg/ml bile (Sigma,St. Louis, MO, USA)), 40 mM sodium bicarbonate (Sigma), 1% glucose and 10% serum for 2 h or 8 h [11]. Encystation efficiency was assayed by treatment for 30 minutes with 0.1% sarkosyl on ice, which lyses trophozoites, allowing the percentage of mature (>48 h) cysts in the population to be calculated. For early (8 to 24 h) time points at which cysts are not sarkosyl resistant a separate tube of parasites, placed into encystation media at the same time, was allowed to complete development and encystation efficiencies calculated. Excystation efficiency was calculated as percentage of sarkosyl-sensitive trophozoites at 24 h after transfer to excystation media. Nuclear staining was performed using Syto-11 nucleic acid stain (Life Technologies, Foster City, CA, USA) and imaged on a Leica CTR6500 using Leica Application Suite Advanced Fluorescence software.

### RNA extraction and preparation of whole transcriptome sequencing libraries

Two independent biological replicates were generated for each time point for the RNA-Seq libraries; a third biological sample was used to generate RNA for Northern blot analyses. When possible, samples from the same encystation experiment were used for the RNA-Seq libraries. Sample groupings are as follows: Cyst 8h-1 and Cyst 24h-1; Cyst 48h-1 and Cyst 72h-1; Cyst 8h-2, Cyst 24h-2 and Cyst 48h-2; Cyst 72h-2; Excyst 2h-1 and Excyst 8h-1; Excyst 2h-2 and Excyst 8h-2. At each time point, parasites were harvested by chilling on ice, spun down, and washed once in cold phosphate buffered saline solution, pH 7.4. Trophozoites, 8 to 24 h encystation and 2 to 8 h excystation samples were immediately resuspended in 5 ml RNA isolation lysis buffer (Ambion, Austin TX, USA)). Mature cysts (48 h and 72 h) were first treated by incubation for 30 minutes on ice in 0.1% sarkosyl to remove any trophozoites or immature cysts. All samples were lysed using a French press at 400 psi, which lyses >90% of cysts (confirmed by visual inspection) without significant shearing of nucleic acids. Following lysis, RNA was isolated using Trizol reagent (Life Technologies) following the manufacturer's protocol. Total RNA was checked for quality using an Agilent BioAnalyzer.

For preparation of cDNA, 5 µg total RNA was treated with Terminator enzyme (Epicentre, Madison, WI, USA) to degrade uncapped RNAs (60 minutes at 30°C in 20 µl total reaction), followed by heat inactivation for 10 minutes at 65°C. Samples were diluted to 100 µl in 1 × DNAse buffer, and treated with DNAseI (Life Technologies) for 20 minutes at room temperature. Samples were purified using the Ribominus cleanup protocol (Life Technologies) and reanalyzed by the BioAnalyzer to determine the level of mRNA enrichment. First-strand cDNA synthesis, using 30 ng of mRNA-enriched RNA as a template, was performed with a modified version of the SMART protocol (SMART; Clontech, Mountain View, CA, USA; originally described as Capfinder) [106,107]. Adaptors containing the rare asymmetrical restriction sites for SfiI were incorporated into the cDNA using a template switching mechanism at the 5' end of the RNA transcript. For SMART PCR amplification of first-strand cDNA, a SMART PCR primer was used to anneal to identical sequence regions on both the 3' and 5' adaptors (primers used for first strand synthesis are listed in Additional file 12). Following 20 to 24 cycles of PCR amplification using Advantage Taq (Clontech) according to the manufacturer's instructions, samples were digested with SfiI (New England Biolabs, Ipswich, MA, USA) to remove the majority of adaptor sequences. Samples were purified using a Nucelospin column (Macherey Nagel, Duren, Germany) to remove digested adaptors.

Amplified, double-stranded cDNA was used to prepare SOLiD™ fragment libraries according to the manufacturer's protocols (Life Technologies). Briefly, cDNA was fragmented by sonication on a Covaris S2 sonicator (Covaris Inc., Woburn, MA, USA) and end-repaired in preparation for P1 and P2 adaptor ligation. Adaptors were ligated and the samples size selected and amplified by standard PCR. DNA was bound to SOLiD™ P1 beads and amplified by emulsion PCR, followed by enrichment for templated beads. The DNA was 3' modified before deposition on the sequencing slide, ensuring attachment of the beads to the slide. Libraries were sequenced on a SOLiD™ 4 sequencer (Life Technologies) to produce 50 bp reads.

### Mapping of whole transcriptome sequencing libraries to the *E. invadens *genome assembly

To determine gene expression levels, sequencing libraries made from cDNA representing the *E. invadens *transcriptome at time points during encystation and excystation were mapped to the *E. invadens *genome assembly using Bowtie v0.12.7 [43]. Colorspace reads of 50 nucleotides were trimmed to 35 nucleotides (removing one base from the 5' end and 14 from the more error-prone 3' end) and mapped, allowing up to three (colorspace) mismatches against the reference (option '-v 3'). Reads mapping to more than one position in the reference genome were not included in the final alignment (option '-m 1'). For additional analyses to detect unannotated and misannotated genes, full-length reads were also mapped using the Tophat v1.3.2 [46]. The reason for these two independent alignments is that Tophat can identify introns (Bowtie cannot) but tends to map fewer reads overall. Tophat detects introns by splitting reads that do not align to the genome at their full length into segments, mapping each segment separately and using this alignment to identify introns. However, for short single-end reads, as in our data, it can map to more junctions if given a set of already predicted splice junctions to confirm. Therefore, a two-step mapping strategy was used. Initial 'unguided' alignments (with no prior predicted splice junctions) were carried out with each library using default parameters (except for specifying minimum and maximum allowed intron sizes of 40 and 4,000 bp) to define splice junctions. Then, all putative splice junctions were collected together with those predicted by *de novo *gene calling. Finally, 'guided' alignments were carried out, using these predicted splice junctions, with minimum and maximum allowed intron sizes of 40 bp and 4,000 bp and otherwise default parameters. Sequence and quality files from all 14 samples, and final normalized FPKM for each gene are deposited at the NCBI Gene Expression Omnibus (GEO) under accession number [GSE45132].

### Identification and characterization of differentially expressed genes

Bowtie alignments from all time points were used to generate FPKM values for each gene and identify differentially expressed genes using Cufflinks v2.0.1 [51]. Expression levels were normalized using upper quartile normalization and *P*-values for differential expression adjusted for a FDR of 0.01. Gene annotations were from the *E. invadens *genome version 1.3 [108]. A separate Cufflinks analysis was run without a reference annotation to identify potential unannotated genes. Pairwise comparisons between each of the seven time points were performed (Additional file 9). GO terms were retrieved from AmoebaDB [58]. Pfam domain analysis was carried out by searching the Pfam database [109] with protein FASTA files downloaded from AmoebaDB [58].

### Defining temporal gene expression profiles

Gene expression profiles over the course of encystation and excystation were defined using the Short Time-Series Expression Miner (STEM) [50]. FPKM expression values were used to define two time series: encystation (0 h, 8 h, 24 h, 48 h, 72 h post-encystation) and excystation (72 h cyst, 2 h and 8 h post-excystation). Genes with FPKM = 0 at any time point were filtered out and each gene's expression values were log-normalized to the first time point, log_2 _(Time point n/Time point 0), to give an individual temporal expression profile. These were clustered into profiles and sets of related profiles as follows. A given number, *x*, of distinct profiles were defined to represent all possible expression profiles over *n *time points (5 for encystation, 3 for excystation) allowing up to a given amount, *y*, of expression change per step. Parameters *x *and *y *were set at 50 and 5 (up to log_2_(32)-fold change per step). Observed gene profiles were assigned to the representative profiles they most closely match. A permutation test (permuting time points) was applied to estimate the expected number of genes assigned to each profile and the observed number of genes assigned is compared to this to identify profiles that are significantly more common than expected by chance. Similar profiles form a cluster of related profiles (color-coded in Additional file 8). GO categories associated with genes were used to test for significant enrichment in profiles and clusters. Significance of GO category enrichment was tested by comparing the number of genes in a profile/cluster of size *s *associated with a GO-category to numbers obtained by randomly sampling the entire gene set with samples of size *s*. The *P*-value, adjusted for testing multiple GO categories, indicates the number of times a random sample contained as many or more genes associated with the same GO category.

### Northern blot analysis

Total RNA was extracted from independent samples of trophozoites, 24 h encysting cells, 72 h cysts and 8 h excysting cells. Total RNA (20 μg) from each was run on a 1% denaturing agarose gel, transferred to nitrocellulose, and hybridized overnight at 68°C with a PCR generated probe labeled with [α-^32^P] dATP to the gene being tested. Primers used for probe generation are listed in Additional file 12.

### Phospholipase D activity and butanol inhibition

PLD activity was measured using the Amplex Red Phospholipase D kit (Molecular Probes, Eugene, OR, USA). Parasites were harvested as trophozoites or at 2 h, 5 h, 10 h, 24 h and 48 h after transfer to encystation media. Immature cysts (2 to 24 h) were resuspended in 1 × reaction buffer, with the addition of 1 × complete protease inhibitor (Roche, Basel, Switzerland) and lysed by freeze-thaw in dry-ice ethanol, while 48 h cysts were pretreated in 0.1% sarkosyl to remove trophozoites and immature cysts, then lysed by sonication into the reaction buffer. Protein concentrations were determined using a Bradford assay, and the same amount of protein per well (either 20 or 30 µg) was used in each assay. Activity was monitored by fluorescence of the Amplex Red reagent at 585 nm, read on a SpectraMax M5 plate reader (Molecular Devices, Sunnyvale, CA, USA). All values were corrected by subtracting the background signal (determined using a negative (no lysate) control) and normalized within each trial to trophozoite lysate activity. At least four independent trials were performed for each time point. For assays using n- and t-butanol, each was added prior to addition of trophozoite lysate (30 µg total protein) to a final concentration of 0.6%; n- or t- butanol was also added to the negative controls to measure background. Three independent trials were performed and each assay normalized to an untreated control, to which no alcohol was added. Mean values and ± standard deviation are shown.

The effect of PLD inhibition on encystation was measured by addition of sterile 0.6% n- or t-butanol (Sigma-Aldrich, St Louis, MO, USA) to the encystation media at the initiation of encystation. Encystation was assayed by parasite survival in 0.1% sarkosyl at 48 h as previously described, and normalized within each trial to the untreated sample. Three independent trials were performed. Mean values and ± standard deviation are shown. *P*-value was calculated using Student's *t*-test.

## Abbreviations

bp: base pair; FDR: false discovery rate; FPKM: fragments per kilobase per million mapped reads; GO: Gene Ontology; HMM: hidden Markov model; JCVI: J Craig Venter Institute; PCR: polymerase chain reaction; PLD: phospholipase D; RNAi: RNA interference; RNA-Seq: RNA-Sequencing.

## Competing interests

The authors declare that they have no competing interests.

## Authors' contributions

Genome assembly and annotation: HL, EC, NH. Cell culture, RNA extraction, and verification of differential expression data: GE. Transcriptome library preparation and sequencing: DW. Mapping and sequence analysis of transcriptome data: GE, GW. Wrote the manuscript: GE, GW, US, NH. All authors read and approved the final manuscript.

## Supplementary Material

Additional File 1 **Flowchart illustrating the JCVI Eukaryotic Annotation Pipeline (JEAP)**. The flowchart illustrates the steps and software used in eukaryotic genome annotation and gene family assignment that were applied to the *E. invadens *genome assembly. Click here for file

Additional File 2 **Putative multi-gene families (of two or more genes) in the genome of *E. invadens*. **Membership of a multi-gene family was defined by sharing the same set of functional protein domains with other genes, rather than by sequence similarity. All genes in the genome are listed, along with membership (or not) of a multi-gene family. In addition, the BLASTP best hit and reciprocal best hit in *E. histolytica *are shown. Click here for file

Additional File 3 **Mapping statistics for all sequence libraries**. Tables recording the total number of reads in each replicate transcriptome library and the total number and percentage of reads aligned to the reference genome sequence. For differential gene expression analysis, Bowtie alignments were of 35 bp reads, allowing up to three mismatches and only retaining uniquely mapped reads (reads that did not align equally well to more than one genome region). For genome annotation-based analyses, Tophat alignments of combined libraries at each time point were of 50 bp reads, using default parameters. The number of introns identified by each alignment was also recorded. Click here for file

Additional File 4 **Read counts, normalized gene expression levels, temporal expression profiles and significant differential expression versus baseline expression for all 11,549 loci**. Table showing expression data for 11,549 annotated *E. invadens *genes. For each gene, the gene ID, product description and genomic location are shown, along with read counts in each gene for each sample, normalized gene expression levels (fragments per kilobase per million mapped reads (FPKM)), lower and upper bounds of the 95% confidence interval ('FPKM_conf_low' and 'FPKM_conf_high') for each time point, temporal gene expression profiles (profile IDs relate to profiles shown in Additional file 8) and significantly differentially expressed genes compared to expression in trophozoites or 72 h cysts (for encystation and excystation, respectively). Click here for file

Additional File 5 **Correlation of read count values per gene among replicates taken at the same time point**. Scatter plots of non-normalized read counts per gene for pairs of replicate libraries per time point. Axes are log-scaled for display purposes. Click here for file

Additional File 6 **Results of BLAST search of the Pfam database using translated unannotated transcripts**. Table showing the results of searching translated open reading frames of putative transcripts that do not overlap annotated genes against the Pfam database to identify unannotated protein coding genes/pseudogenes. Click here for file

Additional File 7 **Validation of annotated introns by transcriptome mapping**. Table recording the status of all 5,894 annotated introns. Predicted introns validated by transcriptome mapping, as well as those where only the 5' or 3' end were validated, are shown. In addition to this, reads mapped entirely within an intron are counted to infer incorrect introns (or incompletely spliced introns). Click here for file

Additional File 8 **Temporal gene expression profiles during encystation and excystation**. Expression profiles during encystation and excystation, estimated by the short time course expression miner (STEM) software. Black lines show representative profiles and red lines indicate individual genes assigned to each profile. Each profile is numbered at the top right (these profile numbers are used in Additional file 4) and a *P*-value indicating the significance of gene enrichment (more genes assigned to profile than expected by chance) is shown at the bottom left. Clusters of similar profiles are indicated by colored shading. Click here for file

Additional File 9 **Results of differential gene expressed analysis for all possible pairwise comparisons**. Genes significantly differentially regulated (FDR <0.01) by Cuffdiff for each of the 42 pairwise comparisons among time points. For each gene, gene ID, locus position, sample 1 name, sample 2 name, sample 1 FPKM, sample 2 FPKM, log fold change (log2(FPKM_2/FPKM_1)), test statistic, uncorrected *P*-value and corrected *P*-value (for FDR <0.01) are shown. Click here for file

Additional File 10 **Complete results of Pfam and GO term analysis**. Worksheet 1 contains all Pfam domains that were significantly (*P *< 0.05) enriched in genes up or down regulated at 8 h, 24 h and 72 h post-encystation, at 2 h post-excystation. Pfam accession number, Pfam symbol, a brief description of the domain, total numbers for each Pfam domain in the *E. invadens *genome, numbers of each domain in the regulated genes, and the *P*-value for enrichment are shown. Worksheet 2 contains all GO terms that were significantly (*P *< 0.05) enriched in genes up or down regulated at 8 h, 24 h and 72 h post-encystation, and at 2 h post-excystation. GO accession number, a brief description, total numbers of genes in each category in the *E. invadens *genome, number of genes in each category among the regulated genes, and the *P*-value for enrichment are shown. Click here for file

Additional File 11 **FPKMs of genes related to meiosis**. Expression of all meiosis-related genes during encystation and excystation. Gene ID, description and FPKM values for each time point are shown for both meiosis-specific and meiosis-associated genes. Click here for file

Additional File 12 **Sequences of all primers used in this study**. Sequences for all primers used in this study. **(A) **Primers used to generate PCR probes used in Northern blotting. For each primer, ID of the targeted gene, primer orientation and sequence are shown. **(B) **Primers used in cDNA first strand synthesis. Primer name and sequence are shown. Click here for file
