# Supplementary figures and images for: The genome and transcriptome of the enteric parasite Entamoeba invadens, a model for encystation
Source: Genome Biol. 2013 Jul 26;14(7):R77. doi: 10.1186/gb-2013-14-7-r77 (PMC4053983; doi:10.1186/gb-2013-14-7-r77)

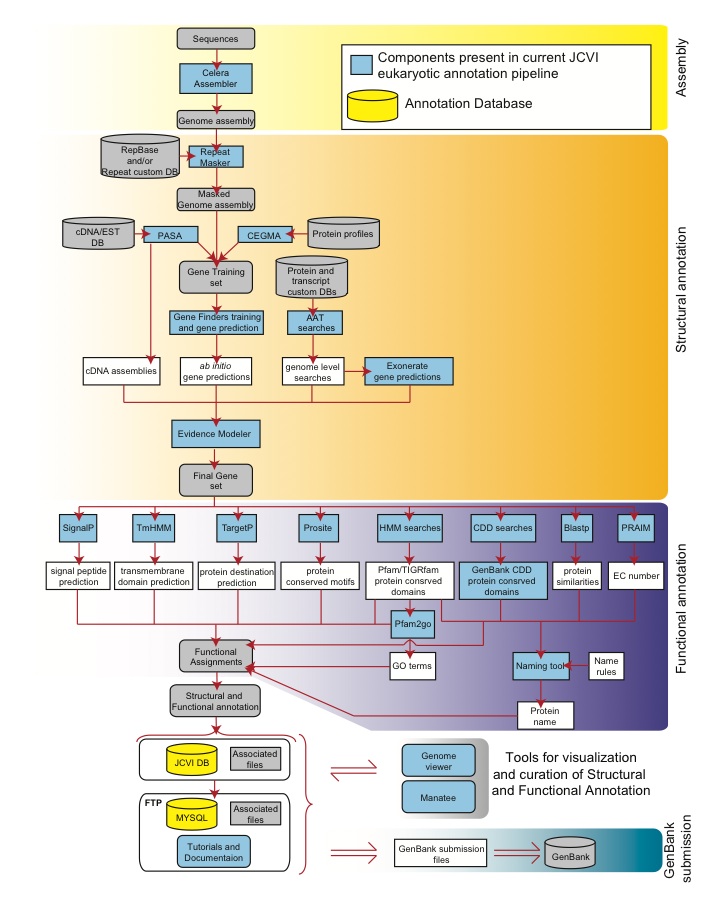

Supplement: Additional File 1 — Flowchart illustrating the JCVI Eukaryotic Annotation Pipeline (JEAP). The flowchart illustrates the steps and software used in eukaryotic genome annotation and gene family assignment that were applied to the E. invadens genome assembly. [file gb-2013-14-7-r77-S1.JPEG]

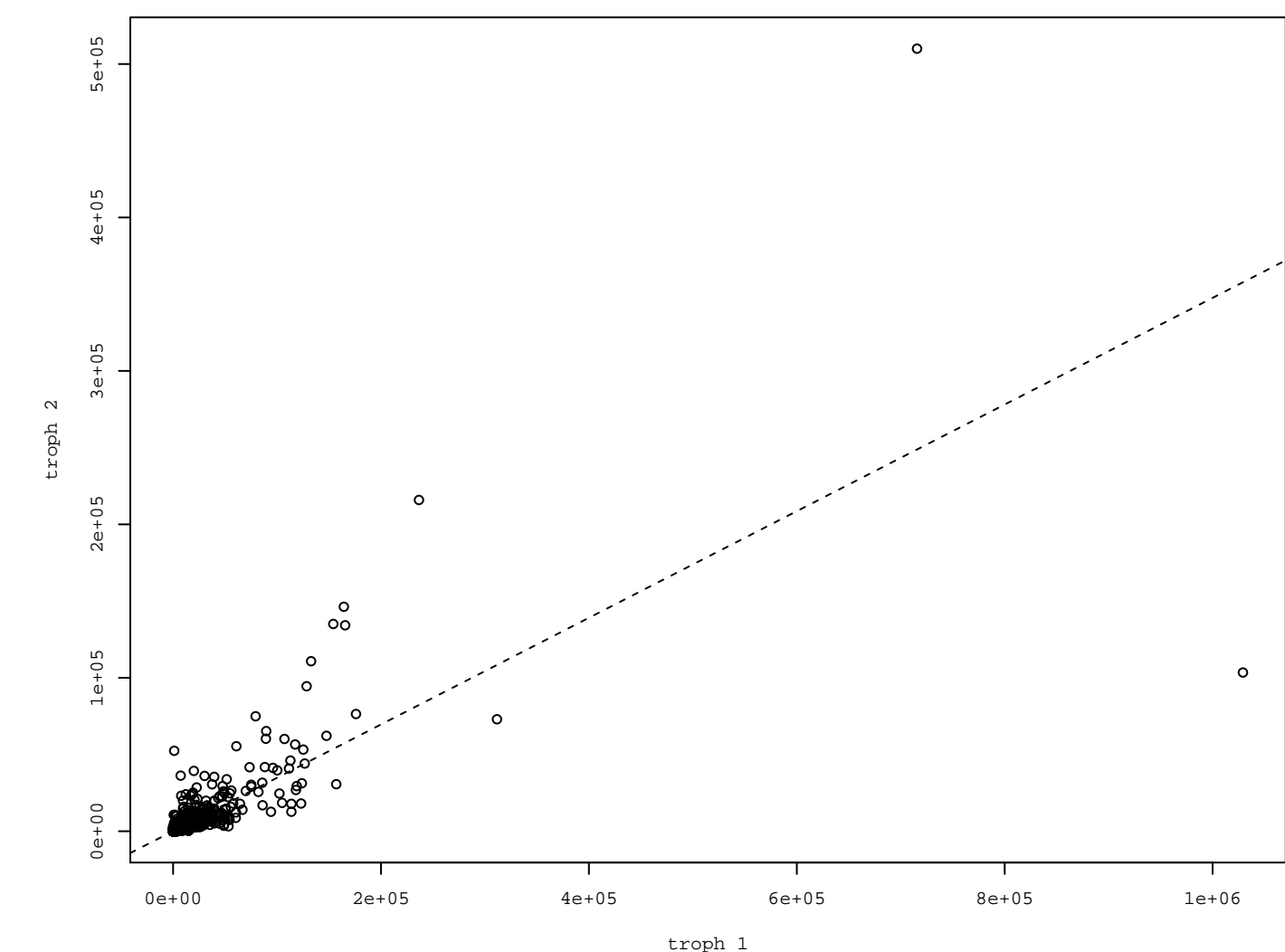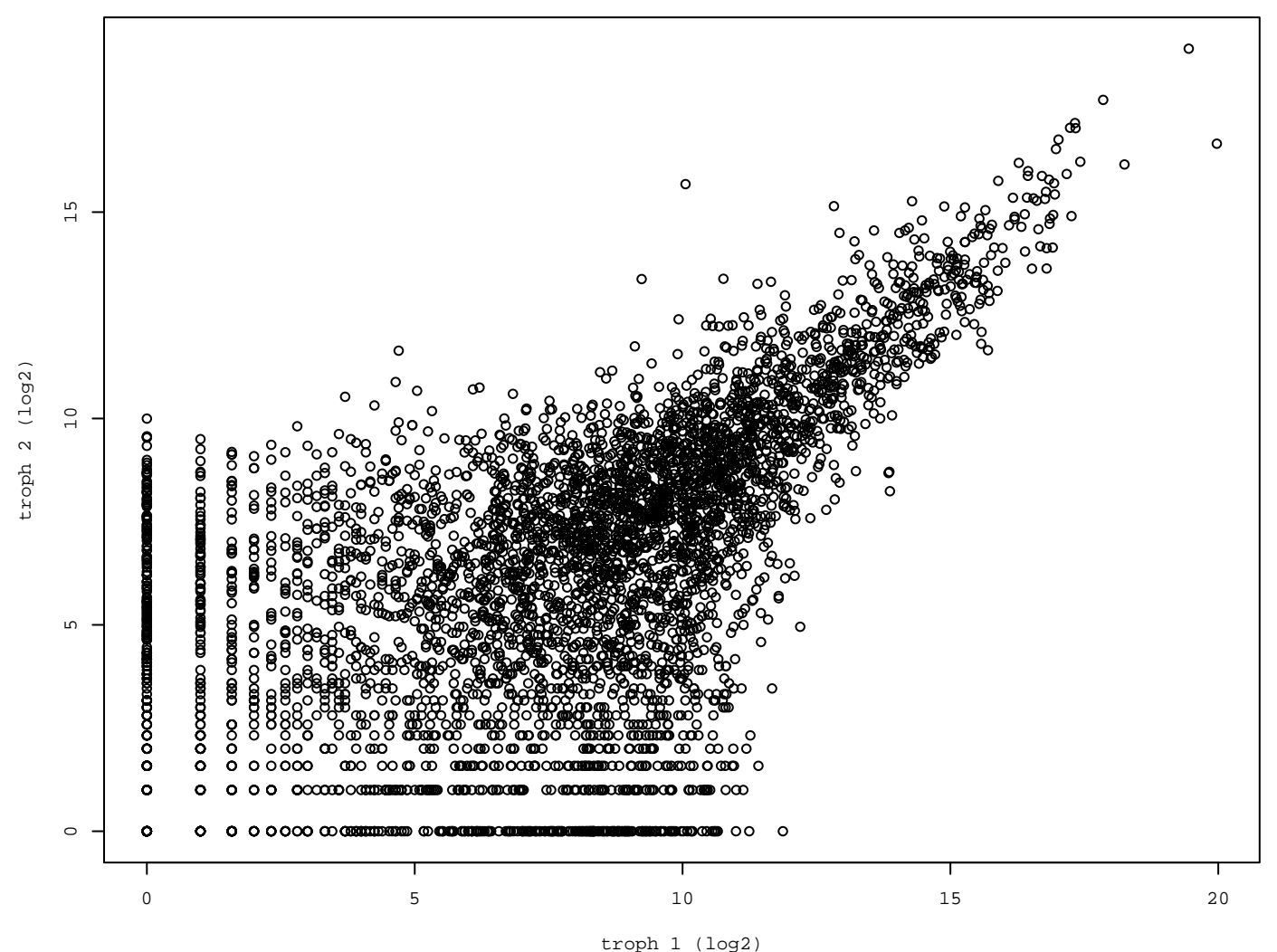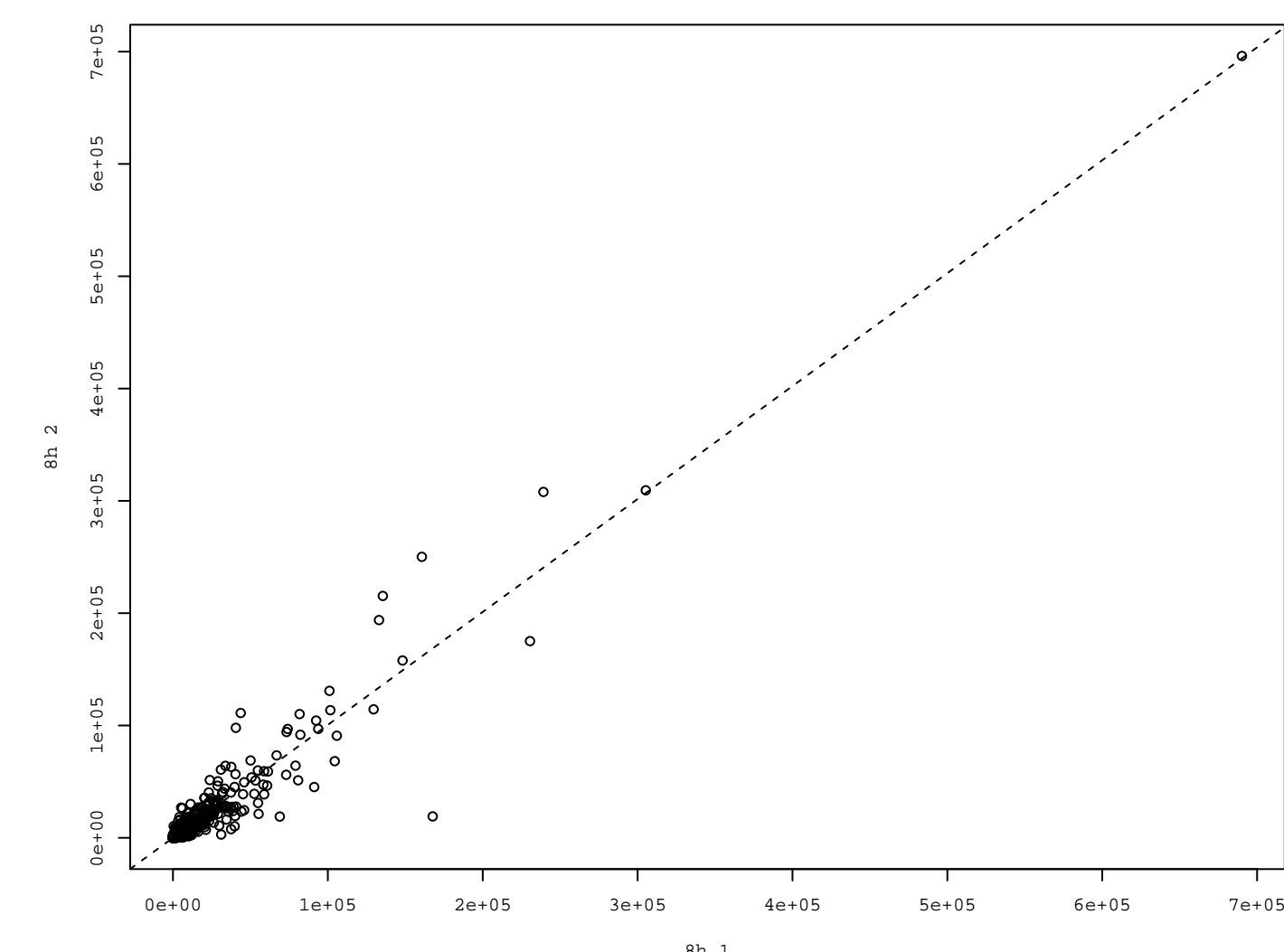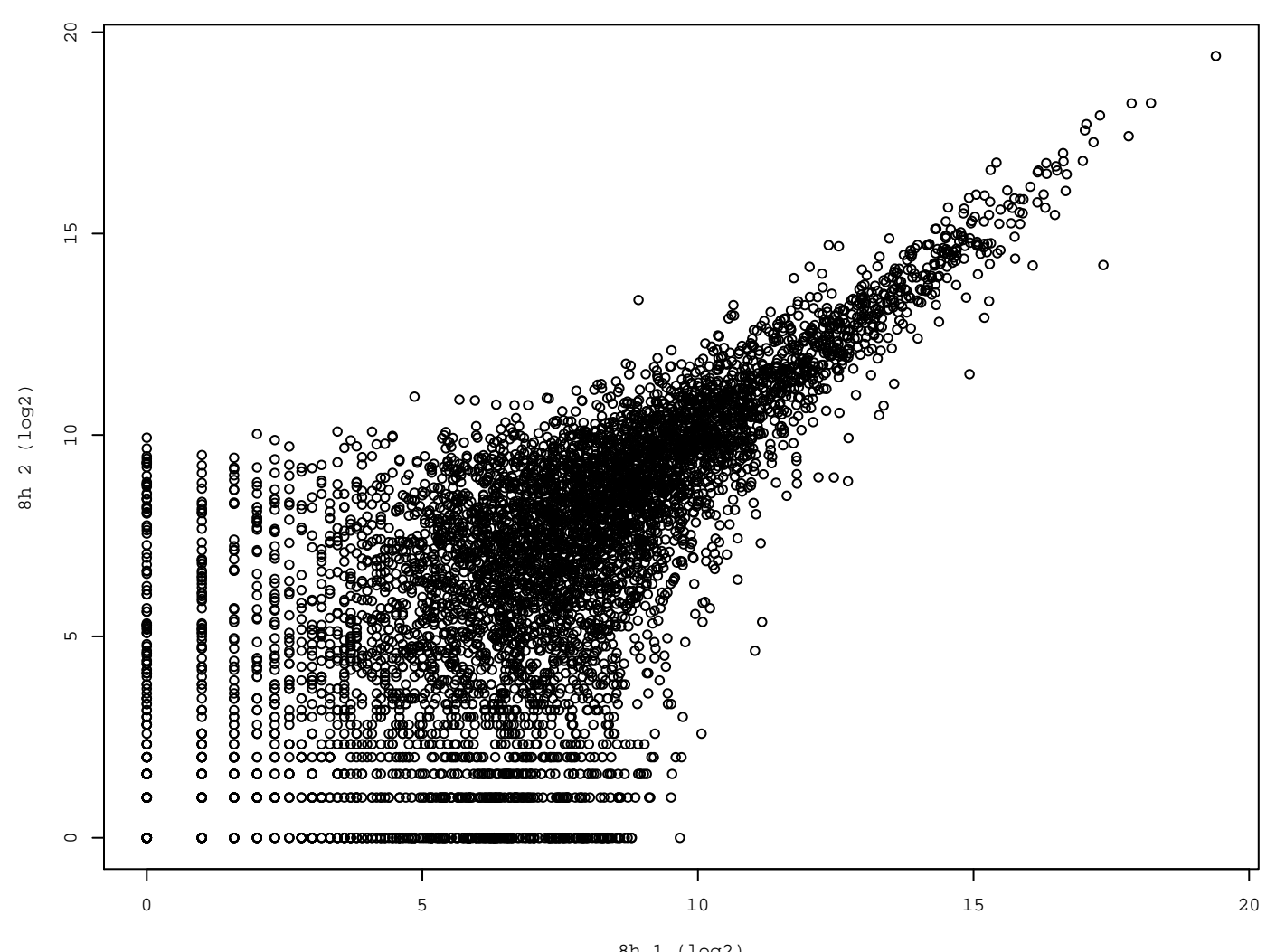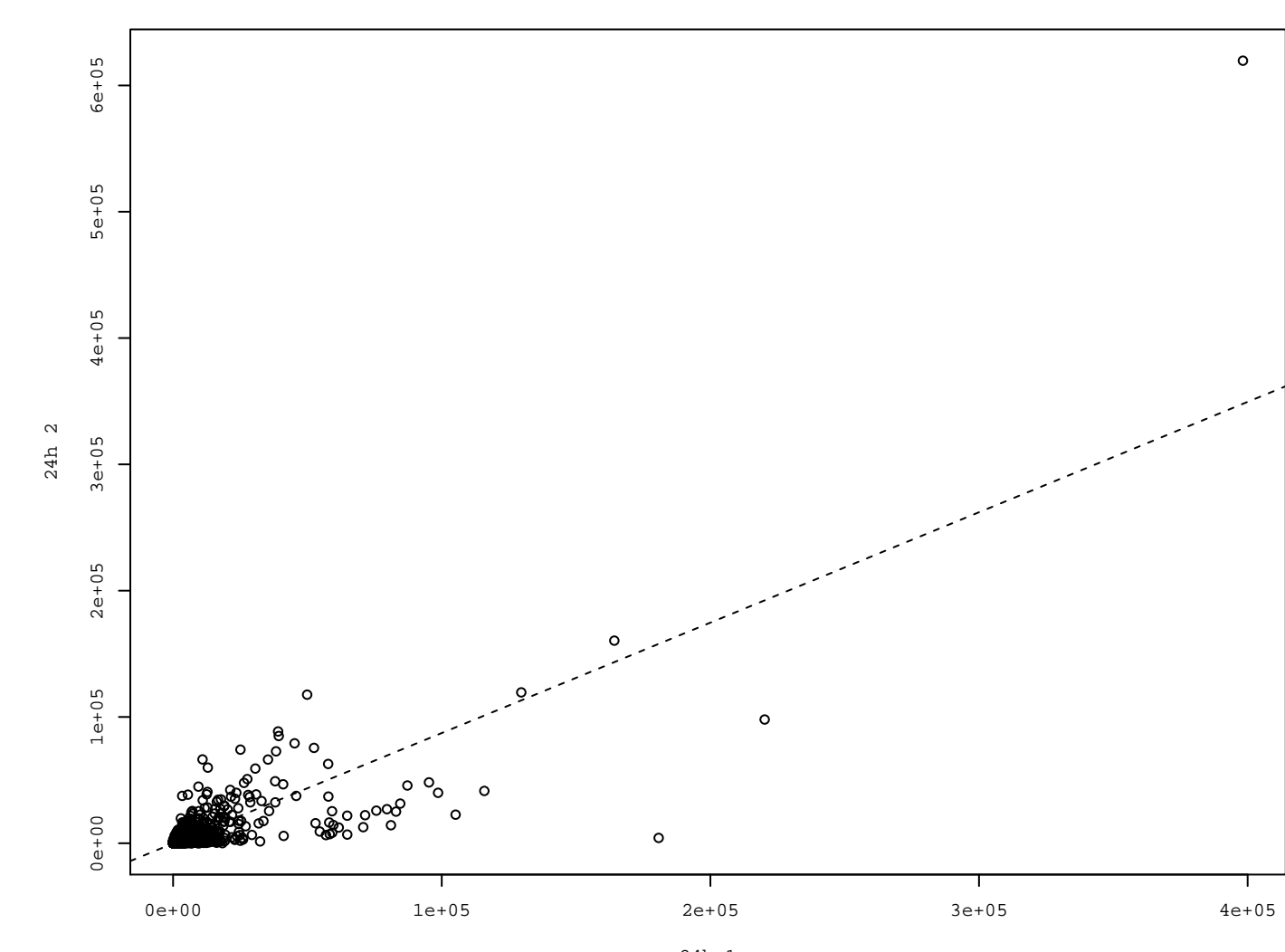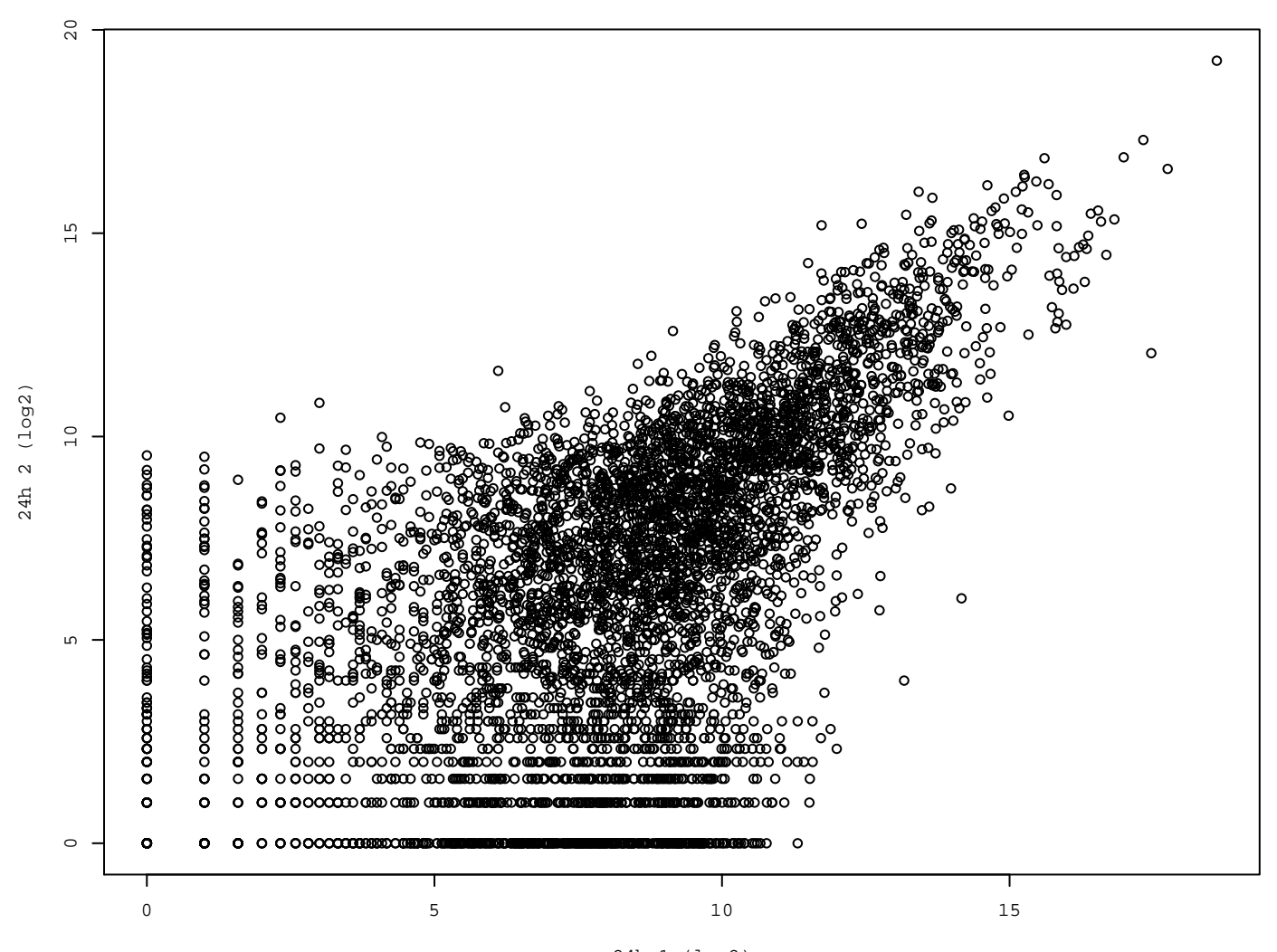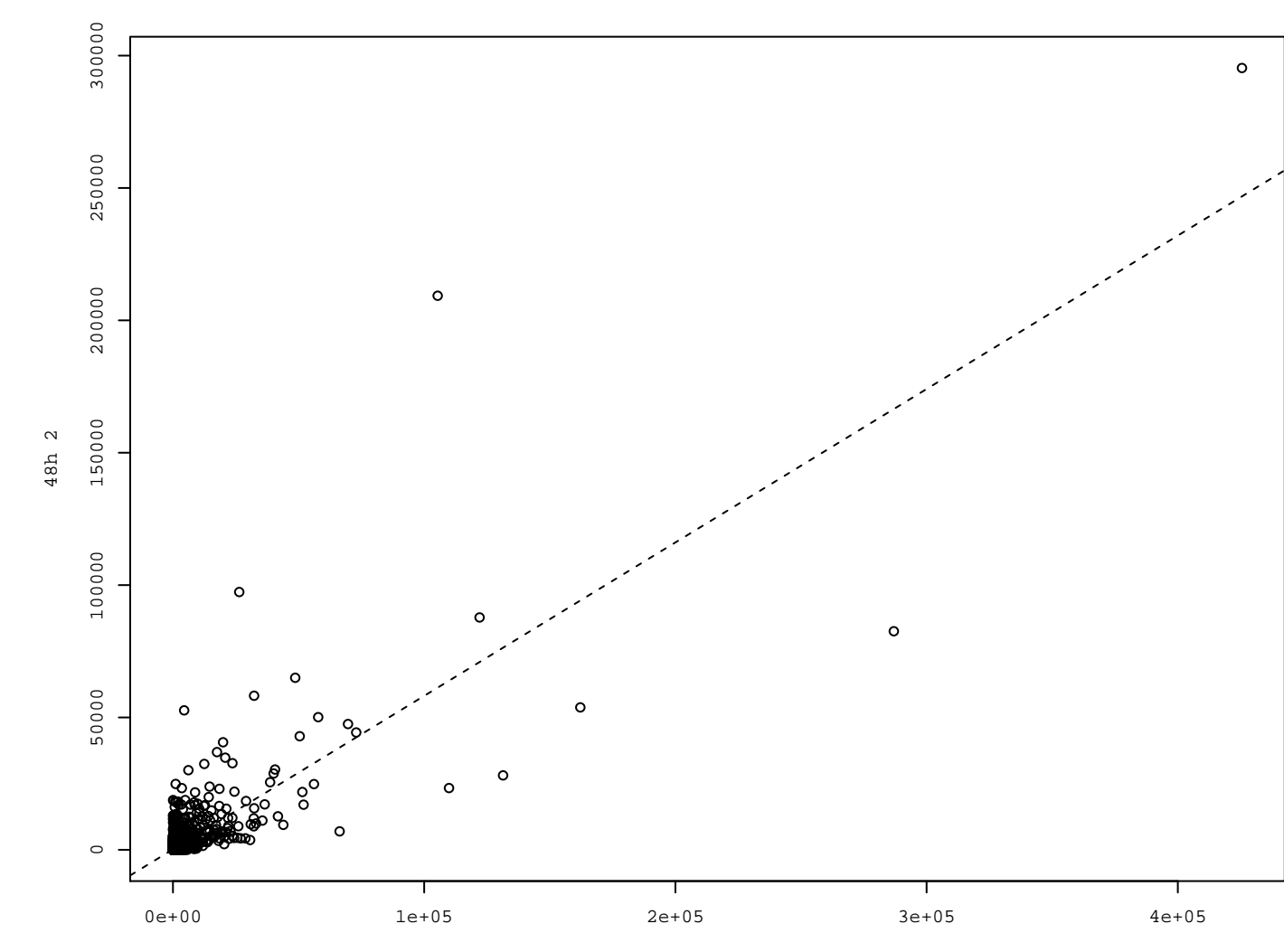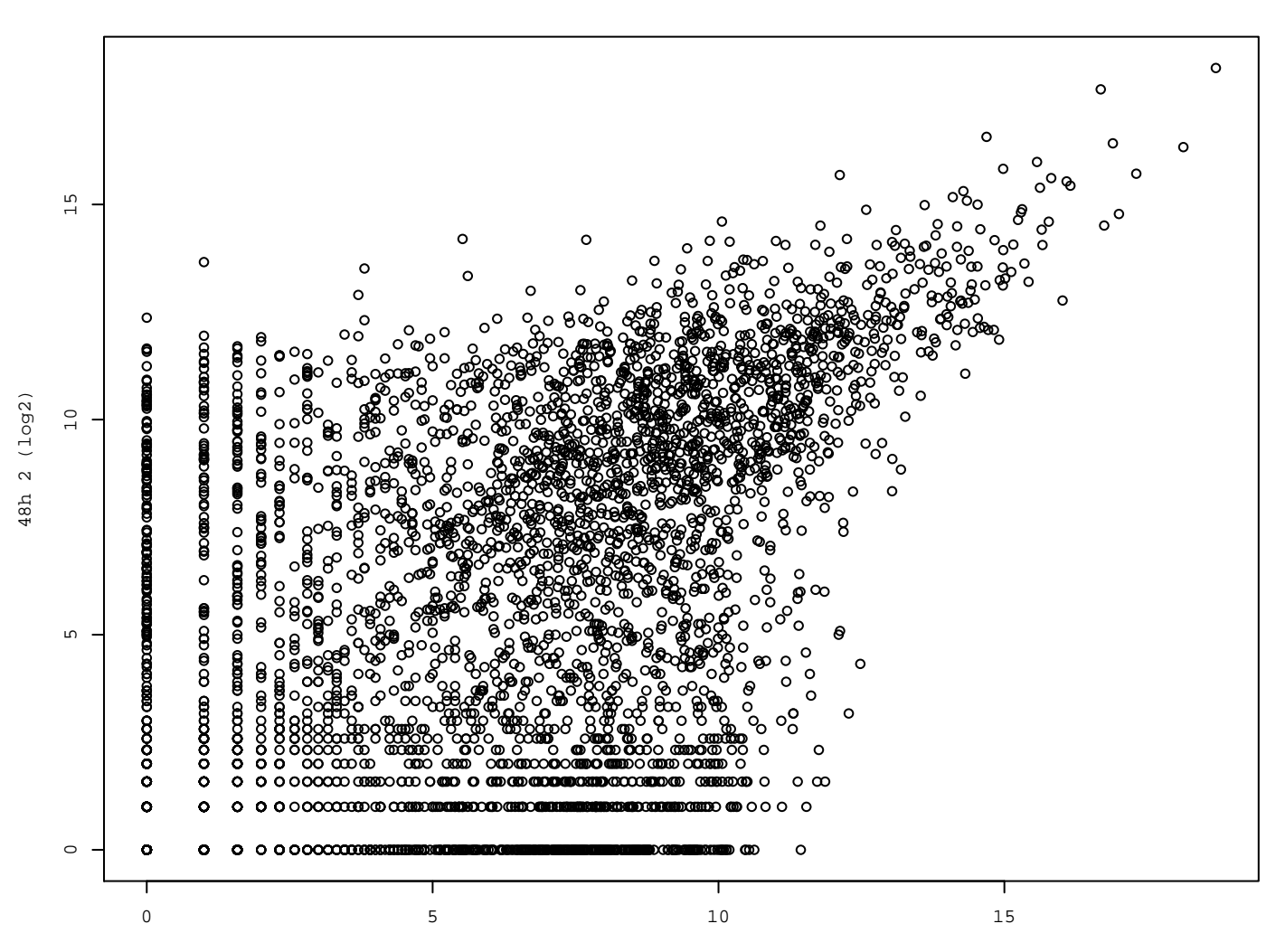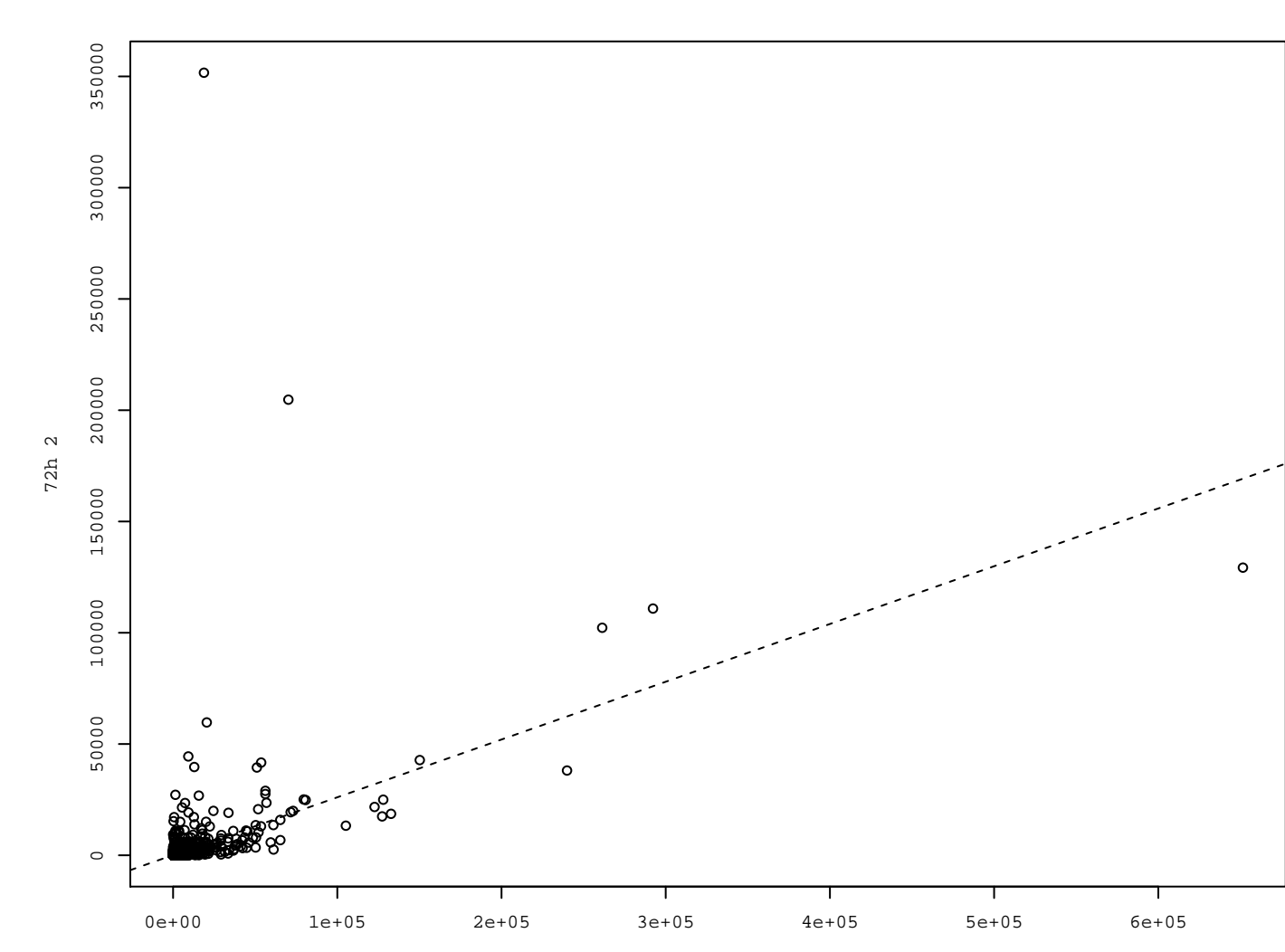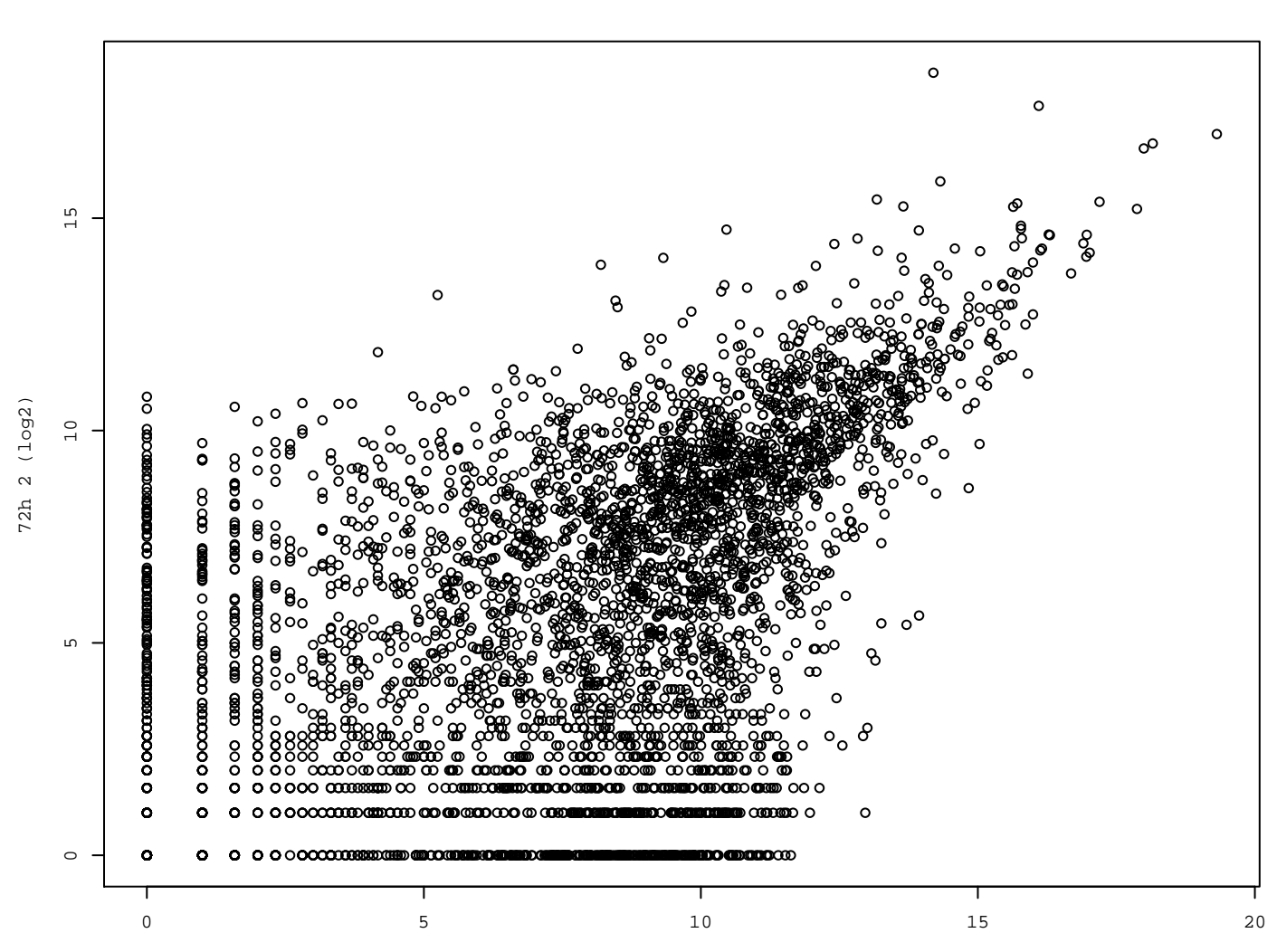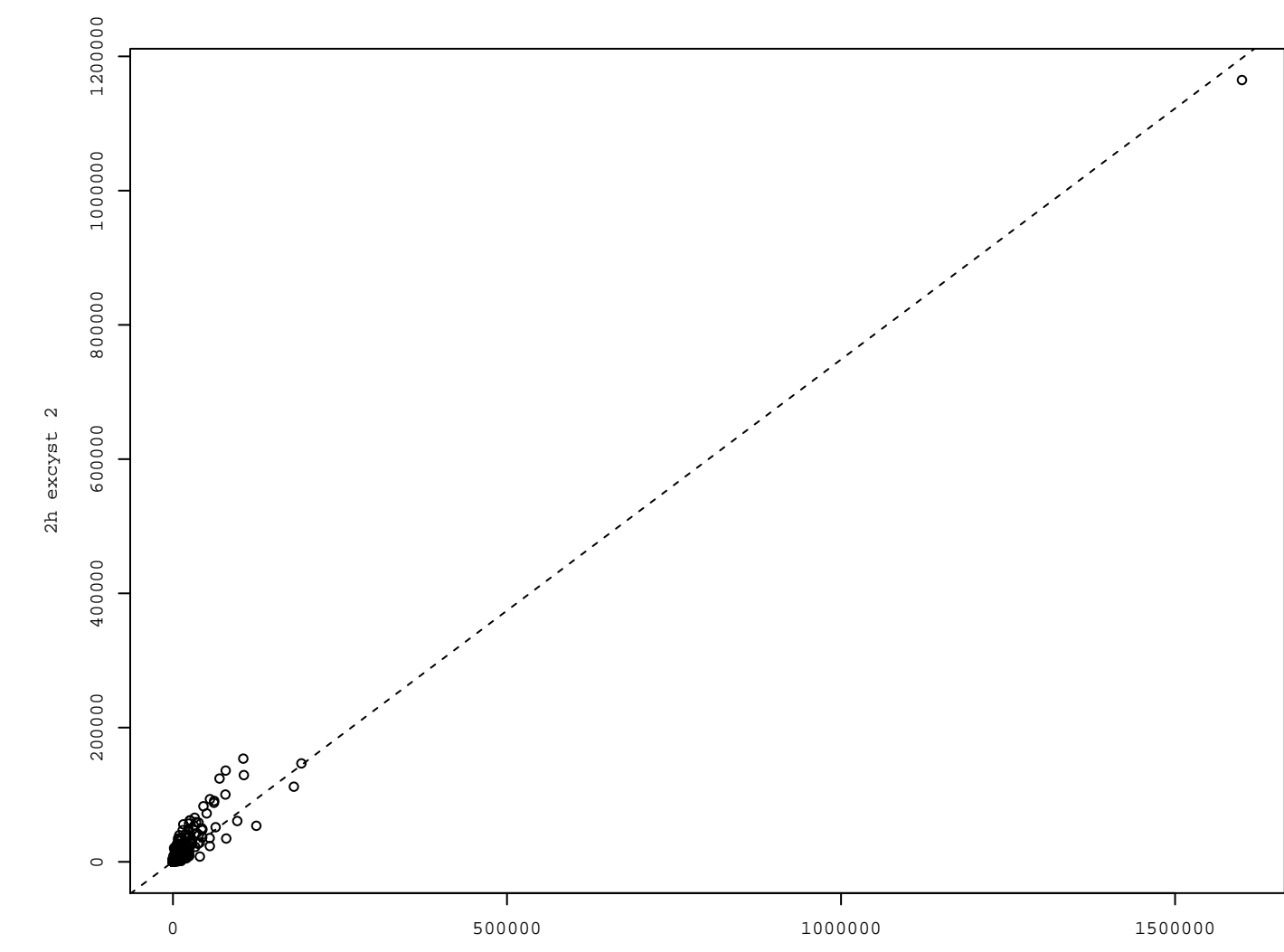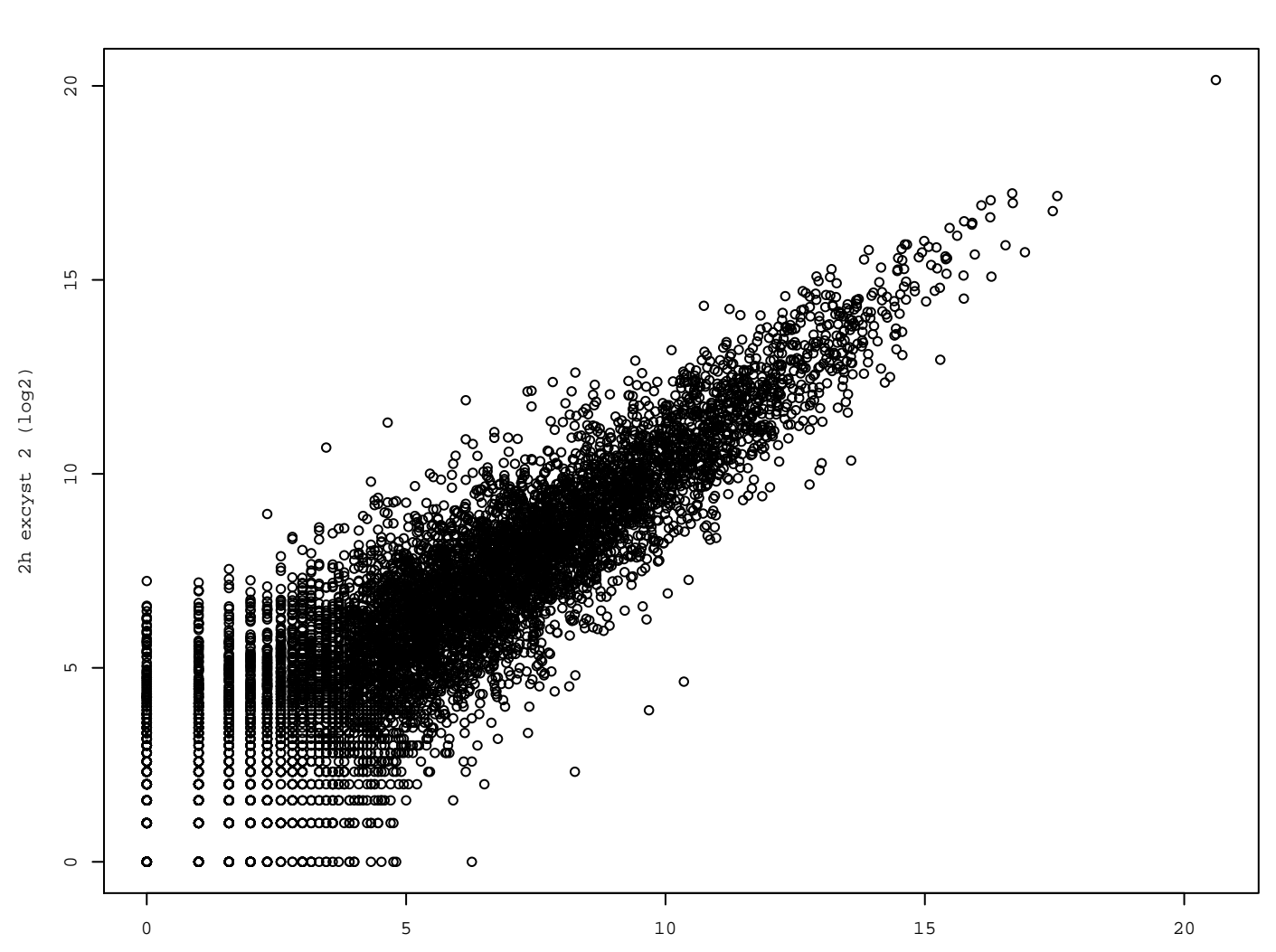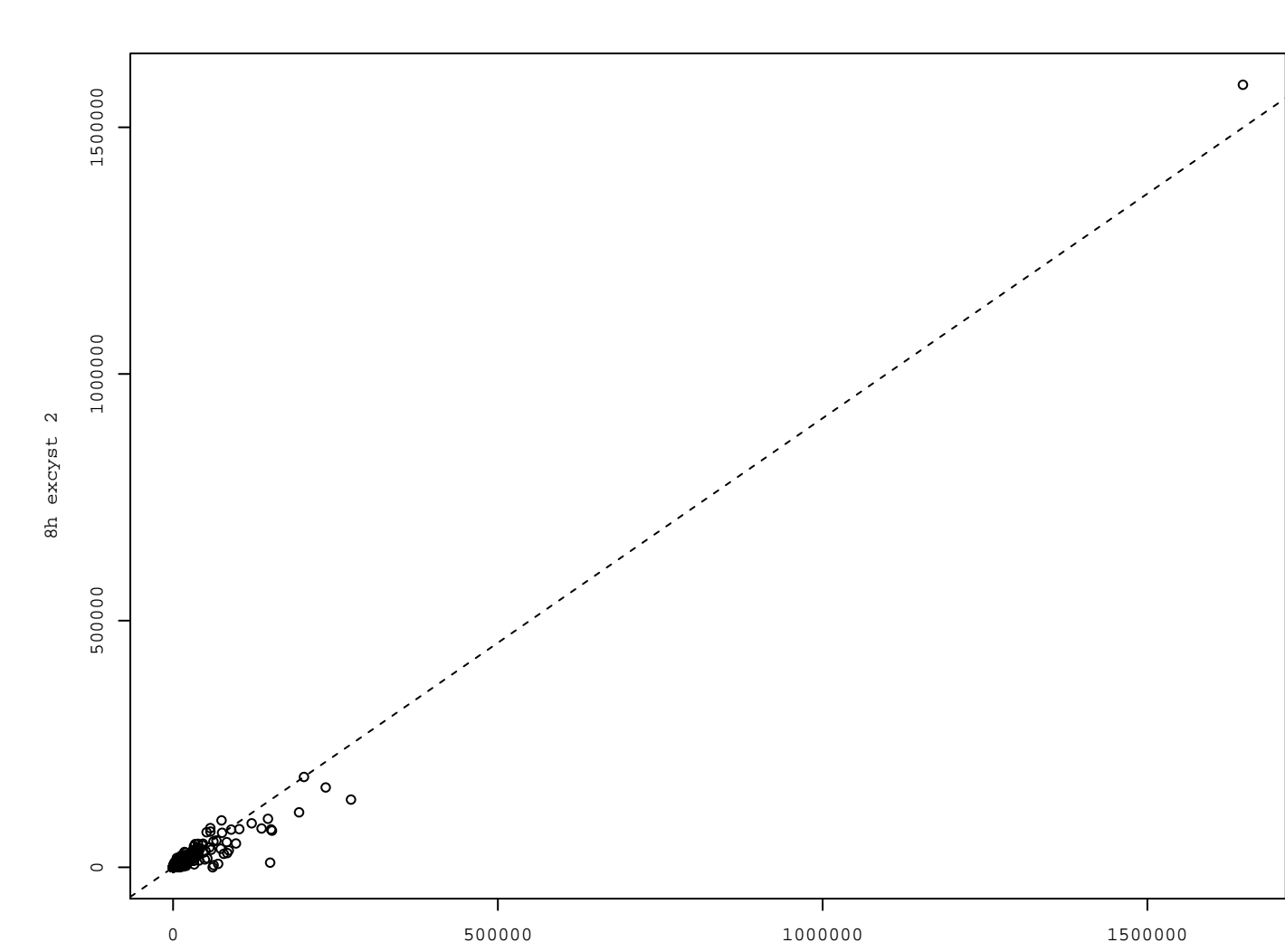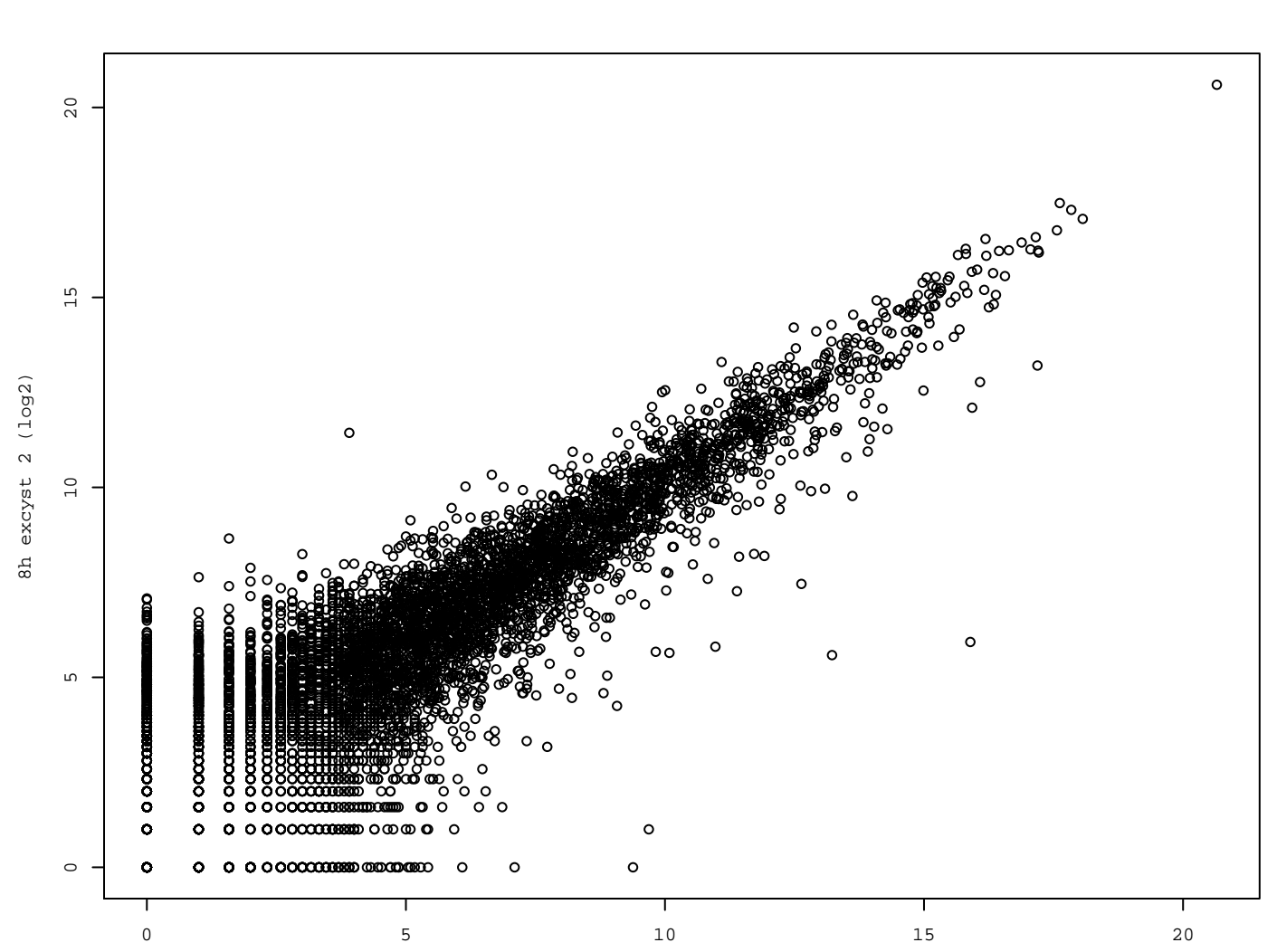

Supplement: Additional File 5 — Correlation of read count values per gene among replicates taken at the same time point. Scatter plots of non-normalized read counts per gene for pairs of replicate libraries per time point. Axes are log-scaled for display purposes. [file gb-2013-14-7-r77-S5.PDF]
