# Supplementary material for: The genome and transcriptome of the enteric parasite Entamoeba invadens, a model for encystation
Source: Genome Biol. 2013 Jul 26;14(7):R77. doi: 10.1186/gb-2013-14-7-r77 (PMC4053983; doi:10.1186/gb-2013-14-7-r77)
Supplement: Additional File 3 — Mapping statistics for all sequence libraries. Tables recording the total number of reads in each replicate transcriptome library and the total number and percentage of reads aligned to the reference genome sequence. For differential gene expression analysis, Bowtie alignments were of 35 bp reads, allowing up to three mismatches and only retaining uniquely mapped reads (reads that did not align equally well to more than one genome region). For genome annotation-based analyses, Tophat alignments of combined libraries at each time point were of 50 bp reads, using default parameters. The number of introns identified by each alignment was also recorded. [file gb-2013-14-7-r77-S3.PDF]

Mapping statistics for Bowtie mappings to measure gene expression levels. Reads were trimmed to 35bp and mapped allowing up to 3 mismatches to the reference genome.

Sample	Stage	Reads_in_library	Reads_mapped_uniquely	%_mapped_uniquely
troph_1	Trophozoite	34805384	18607902	53.46
troph_2	Trophozoite	23643759	7271725	30.76
8h_1	Cyst 8h	29579908	13030952	44.05
8h_2	Cyst 8h	29080575	12895826	44.35
24h_1	Cyst 24h	30623993	12817686	41.86
24h_2	Cyst 24h	28844136	8595653	29.80
48h_1	Cyst 48h	26472132	6882762	26.00
48h_2	Cyst 48h	32552103	6232628	19.15
72h_1	Cyst 72h	24125237	11737489	48.65
72h_2	Cyst 72h	24783665	4167430	16.82
2h_Ex_1	Excyst 2h	24117177	10043890	41.65
2h_Ex_2	Excyst 2h	23989111	11541603	48.11
8h_Ex_1	Excyst 8h	22482562	10816236	48.11
8h_Ex_2	Excyst 8h	18774732	9015519	48.02

Mapping statistics for Tophat mappings to identify novel transcripts and introns. Replicate libraries were combined and untrimmed 50bp reads were mapped to the reference genome with default parameters.

Sample(s)	Stage	Reads_in_library	Reads_mapped	%_mapped	Introns_identified
troph_1 + troph_2	Trophozoite	58449143	20609217	35.26	1628
8h_1 + 8h_2	Cyst 8h	58660483	20635047	35.18	2163
24h_1 + 24h_2	Cyst 24h	59468129	16876073	28.38	1955
48h_1 + 48h_2	Cyst 48h	59024235	10298757	17.45	1047
72h_1 + 72h_2	Cyst 72h	48908902	12573100	25.71	1272
2h_Ex_1 + 2h_Ex_2	Excyst 2h	48106288	7881848	16.38	1438
8h_Ex_1 + 8h_Ex_2	Excyst 8h	41257294	8436142	20.45	764
