# Supplementary material for: The genome and transcriptome of the enteric parasite Entamoeba invadens, a model for encystation
Source: Genome Biol. 2013 Jul 26;14(7):R77. doi: 10.1186/gb-2013-14-7-r77 (PMC4053983; doi:10.1186/gb-2013-14-7-r77)

Temporal gene expression profiles (encystation)

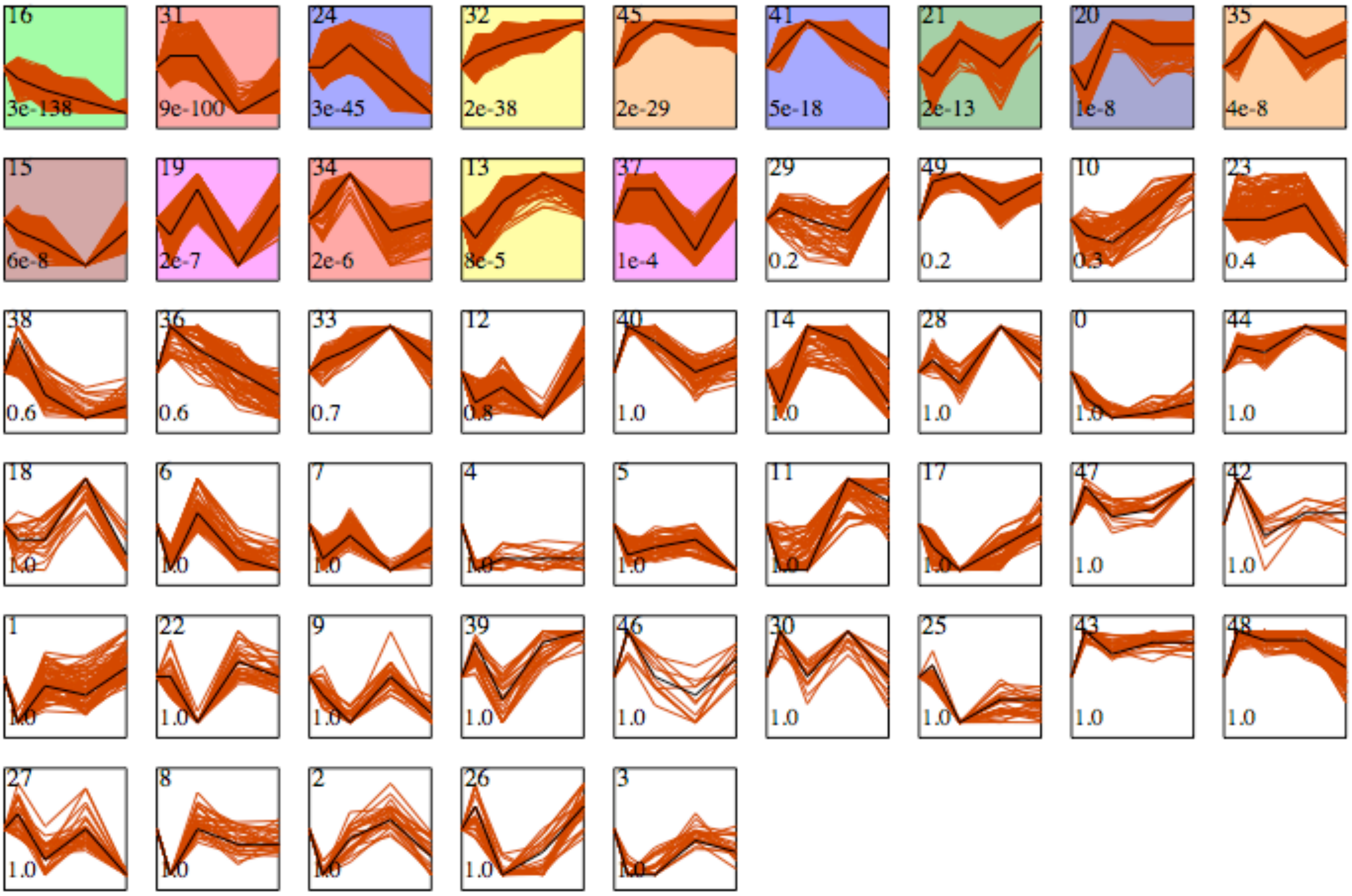

Temporal gene expression profiles (excystation)

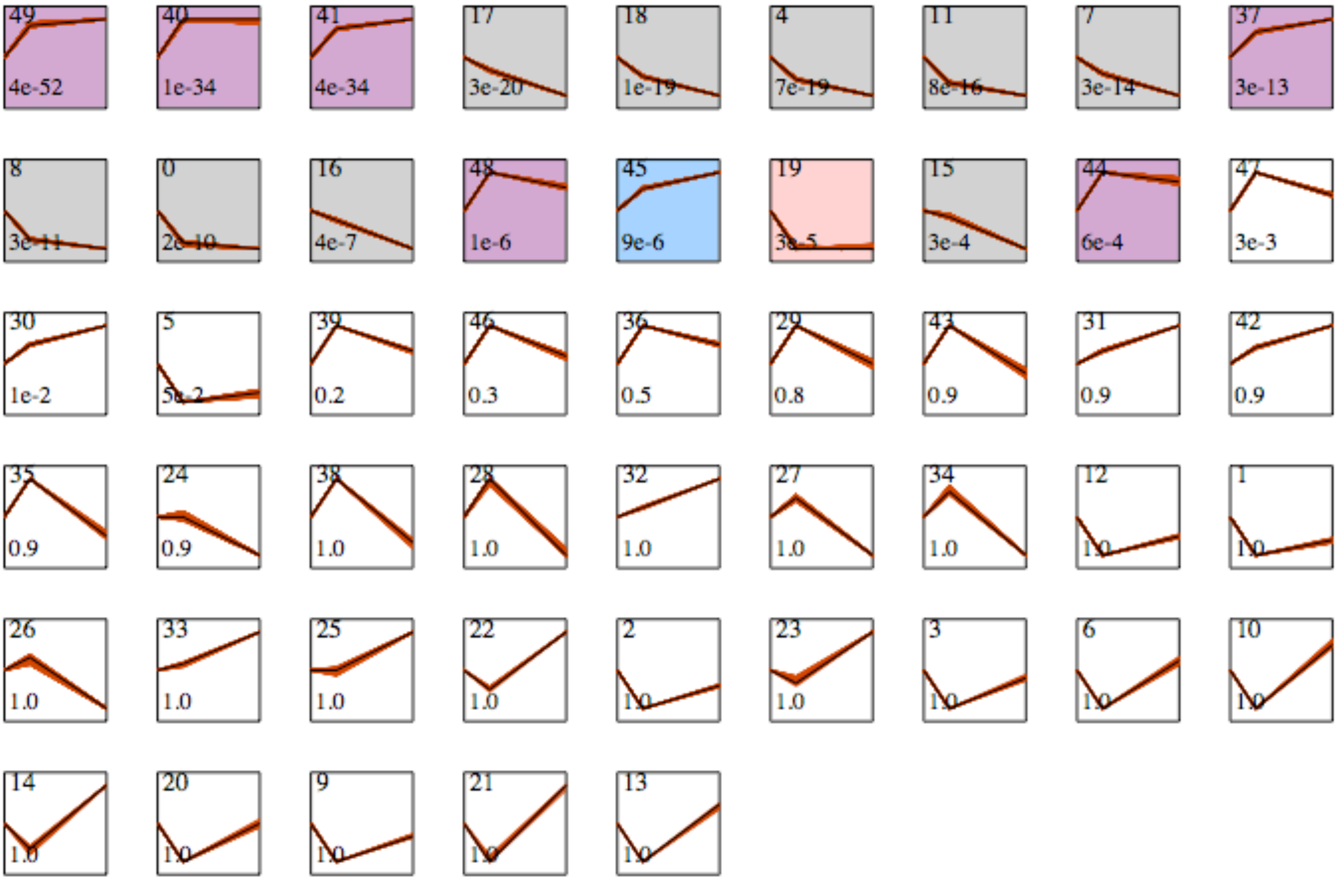

Supplement: Additional File 8 — Temporal gene expression profiles during encystation and excystation. Expression profiles during encystation and excystation, estimated by the short time course expression miner (STEM) software. Black lines show representative profiles and red lines indicate individual genes assigned to each profile. Each profile is numbered at the top right (these profile numbers are used in Additional file 4) and a P-value indicating the significance of gene enrichment (more genes assigned to profile than expected by chance) is shown at the bottom left. Clusters of similar profiles are indicated by colored shading. [file gb-2013-14-7-r77-S8.PDF]
